# Uncoupling of Bacterial and Terrigenous Dissolved Organic Matter Dynamics in Decomposition Experiments

**DOI:** 10.1371/journal.pone.0093945

**Published:** 2014-04-09

**Authors:** Daniel P. R. Herlemann, Marcus Manecki, Christian Meeske, Falk Pollehne, Matthias Labrenz, Detlef Schulz-Bull, Thorsten Dittmar, Klaus Jürgens

**Affiliations:** 1 Biological Oceanography, Leibniz Institute for Baltic Sea Research, Warnemünde (IOW), Rostock, Germany; 2 Marine Chemistry, Leibniz Institute for Baltic Sea Research, Warnemünde (IOW), Rostock, Germany; 3 Research Group for Marine Geochemistry (ICBM-MPI Bridging Group), Institute for Chemistry and Biology of the Marine Environment, University of Oldenburg, Oldenburg, Germany; University of Yamanashi, Japan

## Abstract

The biodegradability of terrigenous dissolved organic matter (tDOM) exported to the sea has a major impact on the global carbon cycle, but our understanding of tDOM bioavailability is fragmentary. In this study, the effects of preparative tDOM isolation on microbial decomposition were investigated in incubation experiments consisting of mesocosms containing mesohaline water from the Baltic Sea. Dissolved organic carbon (DOC) consumption, molecular DOM composition, bacterial activities, and shifts in bacterial community structure were compared between mesocosms supplemented with riverine tDOM, either as filtered, particle-free river water or as a concentrate obtained by lyophilization/tangential ultrafiltration, and those containing only Baltic Sea water or river water. As shown using ultra-high-resolution mass spectrometry (15 Tesla Fourier-transform ion cyclotron resonance mass spectrometry, FT-ICR-MS) covering approximately 4600 different DOM compounds, the three DOM preparation protocols resulted in distinct patterns of molecular DOM composition. However, despite DOC losses of 4–16% and considerable bacterial production, there was no significant change in DOM composition during the 28-day experiment. Moreover, tDOM addition affected neither DOC degradation nor bacterial dynamics significantly, regardless of the tDOM preparation. This result suggested that the introduced tDOM was largely not bioavailable, at least on the temporal scale of our experiment, and that the observed bacterial activity and DOC decomposition mainly reflected the degradation of unknown, labile, colloidal and low-molecular weight DOM, both of which escape the analytical window of FT-ICR-MS. In contrast to the different tDOM preparations, the initial bacterial inoculum and batch culture conditions determined bacterial community succession and superseded the effects of tDOM addition. The uncoupling of tDOM and bacterial dynamics suggests that mesohaline bacterial communities cannot efficiently utilize tDOM and that in subarctic estuaries other factors are responsible for the removal of imported tDOM.

## Introduction

Large amounts of dissolved organic matter (DOM) are transported by riverine waters to coastal oceans [1]–[3], where it becomes an important component of the global carbon cycle [2]. The majority of riverine DOM derives from vascular plants and is thus of terrestrial origin [4]. With warmer temperatures, the export of terrigenous DOM (tDOM) is expected to increase worldwide and especially in subarctic areas, because of melting of the permafrost and an increase in precipitation [5]–[8]. The subarctic and arctic seas already receive large inputs of freshwater and organic matter and the increased export of tDOM due to increasing winter temperatures may influence the aquatic carbon cycle in this region [6], [9], [10].

The bioavailability of tDOM is a determining factor in the aquatic carbon cycle and thus of the potential for feedback effects on global warming. Refractory tDOM is distributed through the global oceans on time scales of decades to thousands of years, whereas labile tDOM provides an important resource for microbial communities in coastal areas, in which case the effects on both higher trophic levels and the global carbon cycle are immediate. This scenario leads to several questions: How much of the imported tDOM is bioavailable [2], [11]? What portion is transferred to the open ocean? And what are the mechanisms regulating these pathways? Arctic tDOM seems to be relatively stable over the time scale of mixing on the Arctic shelves [12]–[15], consistent with only minor losses of tDOM determined in incubation experiments [14]. More recent large-scale studies, however, suggest significant losses of tDOM within the Arctic Ocean [16], [17]. For example, it has been estimated that, globally, about 30% of tDOM is removed during transport across the ocean shelf [18] such that it comprises only a small fraction of the total DOM in the ocean [4], [11].

The microbial decomposition of tDOM has been examined in various types of experiments (see review in [19]). However, published reports investigating DOM decomposition and microbial community composition in parallel are scarce and are usually based on a small number of model substrates (e.g., [20]–[24]). Moreover, the preparative concentration of DOM–such as obtained by the commonly used method of tangential-flow ultrafiltration–also influences the molecular composition of DOM and, in turn, potentially also the decomposition rates [12], [25], [26]. To better understand the bidirectional interaction between tDOM and the microbial community, a parallel, high-resolution analysis of both DOM and the microbial community response is necessary [19], [27].

The objective of this study was to examine the microbially mediated decomposition and the molecular modifications of introduced tDOM and, conversely, the response of a mesohaline microbial community during its exposure to tDOM. To take into account the impact of preparative DOM isolation procedures on tDOM dynamics, mesocosm incubation experiments were carried out with different tDOM preparations (concentrated by ultrafiltration or lyophilization vs. the addition of non-concentrated tDOM after 0.2-μm filtration) mixed with coastal mesohaline water from the Baltic Sea. Water from the Kalix River in Northern Sweden was used as the tDOM source since the geochemistry of its water is comparable to that of large Siberian and Canadian rivers [28]. The Northern Baltic can therefore be considered as representative of Arctic estuaries [29], [30]. We hypothesized that the addition of tDOM and changes in tDOM quality, as a function of the concentration procedure, influence microbial communities and their decomposition activity. Contrary to our expectations, the results showed that bacterial community dynamics were, in general, only slightly affected by the addition of tDOM and over the course of the experiment largely uncoupled from it.

## Materials and Methods

### Sample Collection and Preparation

From mid-May to late June, rivers in the subarctic region reach their peak flow because of melting snow. During this period, discharge from the Kalix River increases sharply, by a factor of 20–30, as the maximum discharge reaches around 1600 m^3^s^−1^ [31]. The catchment (23846 km^2^) drained by the Kalix River is rich in peatland (17–20%), coniferous forest (55–65%) and is sparsely populated (<1% farmland) [32]. For the experiments in this study, Kalix River water was collected from a bridge close to Svartbyn, Sweden (66°15′26.97″N; 22°49′28.38″E) on June 3, 2011, using an *in-situ*-pump module from the Institute for Baltic Sea Research, Warnemünde (IOW) pump-CTD-system [33]. The pump was connected inline to two polypropylene filter holders (MTS40-PP-3/4, MTS Filtertechnik GmbH, Germany), containing a 10-μm filter for prefiltration (TAX10-10, MTS Filtertechnik GmbH, Germany) and a 0.2-μm filter (GR22-PFG-110-002-0, MTS Filtertechnik GmbH, Germany). Measurements before and after sample collection showed that the dissolved organic carbon (DOC) concentration did not change during this process. The filtered river water was transported in 1000-L polyethylene tanks to the Institute for Baltic Sea Research, Warnemünde (approximately 48 h transport), where it was distributed into 25-L polyethylene carboys that were then stored at −20°C until use.

For the experiment, the stored, frozen water was thawed and separate aliquots of the 0.2-μm-filtered tDOM were concentrated by either tangential-flow ultrafiltration or lyophilization [34]. In the former, 90 L of Kalix River water was filtered through a 1-kDa Omega polyethersulfone UF membrane (Pall, Germany) according to the set-up of Brockmeyer and Spitzy [35]. The resulting 3.3 L of ultrafiltrate had a DOC concentration of 4408 (±24) μmolC L^−1^ (recovery rate 40%). For lyophilization, 3.6 L of Kalix River water was lyophilized for 3 days until no ice was seen inside the bottles. The lyophilisate was dissolved in 200 ml of ultrapure water to a final concentration of 4703 (±22) μmolC L^−1^. The recovery rate of 64% was rather low for the lyophilization and may have been due to incomplete resolution of the organic molecules from the freeze-dried material or to the loss of volatile substances. Alternatively, 40 L of Kalix River water was filtered through a second 0.2-μm-mesh filter and the filtrate added directly as natural, unconcentrated tDOM. All glassware was combusted at 400°C for 4 h. All non-glass materials were rinsed before use, first with ultrapure water and then several times with sample water.

### Experimental Set-up

Surface coastal Baltic Sea water for the mesocosms was collected on June 22, 2011 approximately 1 km offshore of Warnemünde (54°11′22″N; 12° 4′45″E), by lowering polyethylene carboys (25 L) into the surface water. The water was filtered through a 100-μm net to eliminate larger zooplankton and particles whereas most of the microbial community was retained. Sampling took place during a period of low phytoplankton biomass in this region [36]. No specific permission is required for sampling in this area and no endangered or protected species are involved in the study.

**Figure 1:**
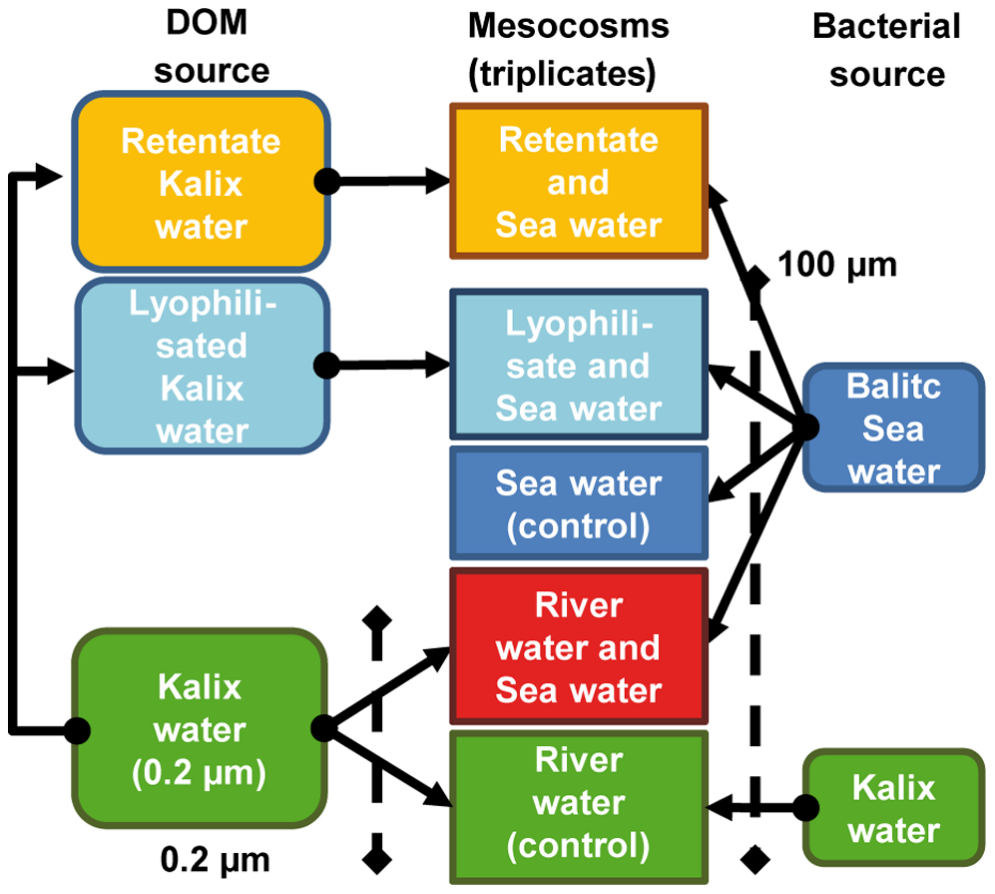
Experimental set-up of the mesocosms. Pretreated tDOM from the Kalix River was mixed with Baltic Sea water and the salinity was adjusted by the addition of artificial sea water and MgSO_4_ (see Tables S1 and S2 for details). The manipulated mesocosms consisted of Baltic Sea water+tangential ultrafiltration retentate (ULTRA), lyophilized Kalix River water+Baltic Sea water (LYO), and Baltic Sea water +0.2-μm-filtered Kalix River water (RB). In the control mesocosms, pretreated Kalix River water was replaced by untreated Baltic Sea water (cBS) and untreated Kalix River water (cRW).

Mesocosms were prepared by mixing pretreated tDOM and Baltic Sea water in five treatments (three replicates each) according to the following scheme (Fig. 1): Baltic Sea water +river water ultrafiltrate (ULTRA), Baltic Sea water+lyophilized river water (LYO), and Baltic Sea water+particle free, unconcentrated river water (RB). Control mesocosms consisted of untreated Baltic Sea water (cBS), particle-free (0.2-μm-filtered) river water (RB)+unfiltered river water (cRW). The volume of each mesocosm was 25 L with the exception of the LYO treatment and the cRW controls, in which the volume was 5 L because lyophilization could not yield a larger quantity of tDOM. Before the addition of 100 μm-filtered Baltic Sea water to the mesocosms, the salinity of the pretreated Kalix River water was adjusted to the *in situ* salinity of 8.1 using 100×-enriched artificial seawater based on Difco Marine Broth 2216 but without organic substrates (Table S1, Table S2). In all experiments, 10 μM NO_3_NH_4_ and 1 μM NaHPO_4_ were added to avoid inorganic nutrient limitation. Dissolved inorganic phosphate, nitrite, nitrate, ammonium, and silicate were measured according to standard photometric methods [37], [38]. The samples were filtered through pre-combusted Whatman GF/F filters and stored at −20°C until analysis, except the sample for ammonia analysis. For the phosphate, nitrite, nitrate, and silicate analyses a continuous-flow analyzer (FLOWSYS, Alliance Instruments) was used. The ammonium content of the samples was determined directly after sampling using a UV mini 1240 photometer (Shimadzu).

The mesocosms were held at a constant temperature of 10°C in dark climate chambers. The pH and ionic strength in each treatment were permanently controlled using pH (WTW pH197i) and conductivity (Haach-Lange CDC401-01) electrodes, respectively. The values did not change significantly during the incubation. Although our intention was to have comparable DOC concentrations in all mesocosms, this was not entirely possible since the natural DOC concentrations of the control mesocosms Baltic Sea (cBS) and Kalix River (cRW) differed (310 and 400 μM, respectively). Thus, the final DOC concentrations in the experimental treatments were in the range of 310–380 μM. Samples were taken, after shaking the carboys, from the 25-L mesocosms (cBS, RB, ULTRA) on days 1, 2, 6, 8, 14, 21, and 28 and from the 5-L mesocosms (LYO, cRW) on days 1 and 28, by decanting a sufficient amount of water in a large beaker from which exactly measured subsamples for the different analyses were taken.

### Dissolved Organic Carbon Analysis

Water samples were filtered through pre-combusted (400°C, 9 h) Whatman GF/F filters into pre-combusted glass ampoules, which were fire-sealed and frozen at −20°C directly after sampling. The samples were melted in an ultrasonic bath, acidified with HCl (30%, Suprapur, Merck, Darmstadt) to pH 2, and then analyzed in a Shimadzu (TOC-VCPH and TNM-1) analyzer by high-temperature catalytic oxidation. The detection limit of the DOC analysis was 5 μM C. Procedural blanks, produced by subjecting ultrapure water to the same procedure as the samples, had an average DOC concentration of 9–10 μmol C L^−1^ (±4 μmol C L^−1^, N = 6). The accuracy of our analysis was determined with the standards QNU202 and QNU204 (511 and 238 μmol C L^−1^, respectively) from a QUASIMEME laboratory (http://www.quasimeme.org/). In all measurements, the difference between the known QNU202 and QNU204 concentrations and the measured one was always <4 and <8 μmol C L^−1^ (2.5 and 3.5% difference, respectively). The average aberration for both standards was <1%.

### Fourier-transform ion Cyclotron Resonance Mass Spectrometry Analysis

Molecular analysis of DOM was performed by Fourier-transform ion cyclotron resonance mass spectrometry (FT-ICR-MS) on a BrukerSolarix FT-ICR-MS (Bruker Daltonik GmbH, Bremen, Germany) equipped with a 15-Tesla magnet system. Negative electron spray ionization (ESI, BrukerApollo II) mode was used for all samples. For FT-ICR-MS analysis, DOM was purified and concentrated by solid-phase extraction (SPE) [39]. In brief, sample aliquots (140–180 ml) were acidified to pH 2 (HCl 32%; Emsure, Merck, Darmstadt) and gravity-loaded on Agilent Bond ElutPPL 1-g cartridges. The cartridges were then desalted with three cartridge volumes of 0.01 M HCl, dried under a stream of N_2_, and DOM was eluted with 6 mL methanol (MS quality, Merck, Darmstadt). DOC concentrations were measured in aliquots of the methanol extracts after they had been dried for about 20 h at 50°C and re-dissolved in ultrapure water. The DOC extraction efficiency was on average 80% (±7%, N = 78). For the procedural blanks, ultrapure water was also processed by SPE. The DOC in these blanks was below the detection limit. Molecular traces in these blanks that were detected by FT-ICR-MS were subtracted from the sample results and thus did not influence the statistical analyses. For FT-ICR-MS analysis, the extracts were diluted to yield a DOC concentration of 20 mg C L^−1^ in methanol and water (1∶1 v/v) and filtered through a 0.2-μm PTFE syringe filter (Rotilabo, Roth, Germany).

**Figure 2:**
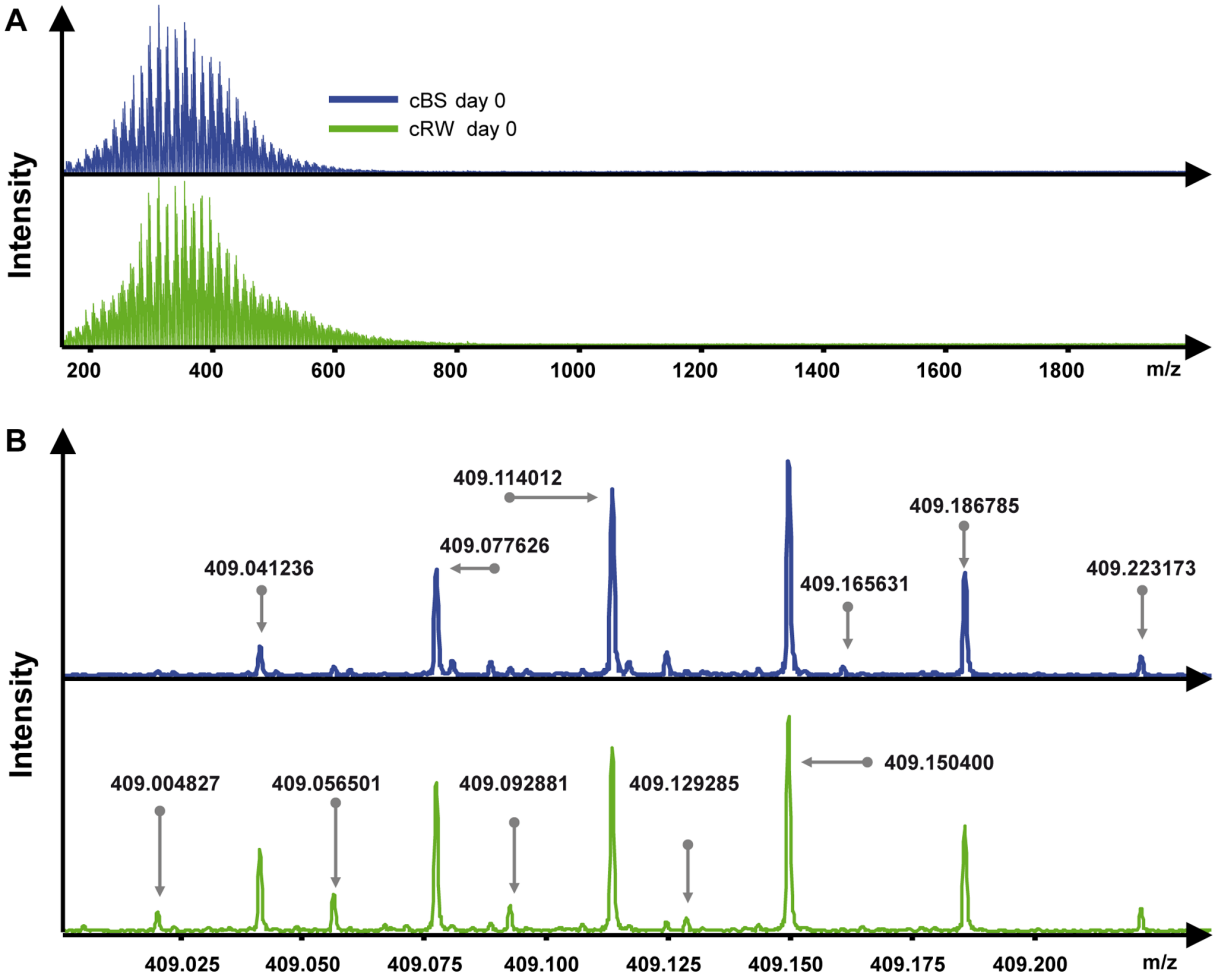
Fourier-transform ion cyclotron resonance mass spectrometry (FT-ICR-MS) mass spectra. (A) Mass spectra showing the complete mass range, from 150 to 1800 Da, covered by FT-ICR-MS. In our samples there were no peaks with a mass to charge ratio above 680. The spectrum of the cBS day 0 samples is colored blue and that of the cRW day 0 sample green. (B) Mass spectra showing an example of the mass range from 409.0 to 409.250 Da of the same spectra. The exact masses of some selected peaks are included.

Every sample was calibrated internally according to an in-house database of 68 molecules with known molecular masses. The mass error of the internal calibration was <0.05 ppm. For instance, the mass error of a compound with a molecular mass of 400 Da was <0.0002 Da, which is <1/10 the mass of an electron. At this high mass accuracy, molecular formulas could be assigned unambiguously to all detected masses following the procedure published in Koch and Günther [40] and by applying the following rules for the number of respective elements: O≤C, N<O, N≤4, S≤2, P≤1; combinations of the elements N, S, and P were not considered. The samples were measured in a mass range from 150 to 1800 Da (Fig. 2). Masses below 150 Da cannot be detected by the setup of the FT-ICR-MS and masses above 680 Da were very rarely observed. Multiple charged ions did not occur. In total, across all samples, we detected 25,226 masses with peak intensities at least three times the noise. We determined, on average, 10,423 different molecular masses per sample and were able to assign 5,933 molecular formulas to these masses, not including isotopologues (monoisotopic peaks containing the isotopes ^13^C, ^15^N, ^18^O, and ^34^S). Their inclusion would have increased the assignment rate considerably. Because of the low signal to noise ratio chosen as the minimum threshold, the data still contained noise peaks. Instead of simply increasing the signal to noise threshold, we applied two alternative procedures to eliminate noise. Both were based on the observation that detected masses of noise peaks are not reproducible, *i.e.*, rarely detected more than once in replicate analyses. First, we considered only those masses that were detected in all triplicates of one treatment, *i.e.*, masses detected in only one or two of the three parallel mesocosms were excluded. Applying this criterion we observed, on average, 9,384 molecular masses per sample and 5,804 molecular formulas. Nonetheless, this data set still contained some noise peaks that may negatively affect the statistical analysis (Figure S1). As an alternative data reduction approach, we considered only peaks that were present in >80% of the samples. This procedure is more restrictive compared to the previous one that considers only masses detected in all triplicates of one treatment. On the other hand, it is also more neutral in that it does not assume *a priori* that replicate mesocosms are more similar than different treatments, which is a major advantage in the context of this study. With this approach, on average 6,007 masses and 4,631 molecular formulae were detected per sample. Multivariate statistical analysis with the two data sets obtained via the two noise reduction procedures revealed only minor differences with respect to treatment comparison. The complete list of assigned DOM compounds is found in Table S3. A direct comparison of significantly changed intensities of molecular masses from day 0 to day 28 was also performed (Figure S2). The day 0 samples of cBS and RB were tested (Student’s t-test, paired, p-value<0.05) against the day 28 samples and visualized in a van Krevelen diagram. For comparison, six artificial samples were generated with randomly set molecular intensities and the first three random samples were tested against the last three random samples (Figure S2C).

### Bacterial Production, Respiration, and Carbon Demand

Bacterial production (BP) was estimated using ^3^H-labeled thymidine incorporation [41]. The radiotracer was added to 4-ml subsamples (3 replicates; final concentration 10 nM), which were then incubated for 1–1.5 h at 10°C in the dark. Incubations terminated by the addition of formaldehyde (10% v/w) were filtered onto 0.2-μm pore size polycarbonate filters, rinsed twice with ice cold 5% trichloroacetic acid, and placed in 5-ml scintillation vials to which 4 ml of scintillation cocktail (Irgasafe Plus, PerkinElmer Life & Analytical Sciences, Netherlands) was added. A formaldehyde-fixed sample was used as control and its measured radioactivity was subtracted from that of the samples. The empirical conversion factor of 2×10^18^ cells per mol incorporated thymidine L^−1^ was used to estimate bacterial cell production [42] and a conversion factor of 25 fg C cell^−1^ was used to calculate bacterial carbon production [43].

Bacterial respiration (BR) was assessed by measuring oxygen consumption in 100-ml subsamples in oxygen-tight glass bottles incubated at the *in situ* temperature (10°C). Oxygen consumption was measured for 12 h using a Hach Lange HQ40d system equipped with a luminescence-based optical sensor (LDO LDO101) for temperature and salinity compensation. The measured oxygen consumption was integrated over a linear slope and corrected against a parallel measurement of ultrapure water. Respiration rates were calculated from oxygen consumed and converted into carbon respired by applying a respiratory quotient of 0.88 [44]. Bacterial growth efficiency (BGE) was calculated as BGE = BP/(BP+BR).

### Bacterial and Flagellate Enumeration

For bacterial enumeration, 4-ml subsamples were fixed for 1 h with 400 μl of 1% paraformaldehyde and 0.5% glutaraldehyde, shock-frozen in liquid nitrogen, and then stored at −80°C until processing by flow cytometry. Samples were measured on a FacsCalibur (Becton Dickinson) using a modification of the method of Gasol et al. [45]. In brief, a 0.2 μm-filtered SYBR Green solution (2.4 M potassium citrate, 0.2 M dimethyl sulfoxide, and 5 μl SYBR Green) was mixed with 300 μl of sample. The mixture was incubated for 30 min in the dark and then measured for 3 min in a flow cytometer at medium flow rate. The flow diagrams were evaluated using the software CellQuestPro.

Samples for the enumeration of heterotrophic and autotrophic nanoplankton (HNAN <10 μm) were preserved with formaldehyde (2% final conc.), filtered through black 0.2-μm black polycarbonate filters (Whatman GmbH), and stained with 4,6 diamidino-2 phenylindole (DAPI; final conc. 2 μg/ml) [46] for 10 min. At least 30 cells per filter were counted at 630× amplification using an Axioskop 2 mot plus epifluorescence microscope (Carl Zeiss MicroImaging, Germany).

### Bacterial Community Composition

Water samples for DNA analysis were filtered (0.22-μm pore size polycarbonate filters) and the DNA was extracted according to Weinbauer et al. [47]. The 16S rRNA gene sequences of 60 samples from the mesocosms were amplified using the protocol described in Herlemann et al. [48]. In brief, the DNA was PCR-amplified over 30 cycles with the primers Bakt_341F and Bakt_805R, which were complemented with sample-specific 5-bp barcodes. Amplicons were purified using Agencourt AMPure XP (Beckman Coulter, Germany) and sequenced at Eurofins MWG Operon using Roche GS FLX titanium series chemistry.

The resulting sequences were denoised using ACACIA version 1.52 [49] with the settings: quality score 25, k-mer distance 13, statistical significance −9, trim to 330. This process corrected 28,051 of 136,037 sequences and excluded 14,918. Reads longer than 300 bp (excluding primer) and without undefined base calls (N’s) were aligned and clustered into operational taxonomic units (OTUs) using the pyrosequencing pipeline at RDP [50] at a 3% level. The most abundant sequence for each OTU was BLAST [51] searched against a local RDP database (v. 10.18; good-quality score sequences only); the OTU inherited the taxonomy of the best hit if it was ≥95% identical over ≥300 bp. To remove background noise, sequencer reads present only once per library were excluded [52]. Potential chimeras were identified among representative sequences from each OTU by DECIPHER [53] and removed if they had no identical (100%) sequence in the SILVA database [54]. For phylogenetic assignment, quality-checked OTUs (1,493 different OTUs in total) were added to the SILVA 111 Ref tree using the parsimony tool provided in ARB [55]. The complete data of the sample were deposited in the European Nucleotide Archive under accession number PRJEB4004.

### Statistical Analysis

After chimera removal and a quality check, the OTU counts were normalized by converting them into relative proportions. The mass spectrometry intensities of the molecular masses measured in the FT-ICR MS were standardized within every sample by subtracting the average sample intensity from every peak and dividing the result by the standard deviation. This procedure yielded both negative and positive values for the various molecular formulae which, however, did not affect the statistical analysis.

Variations in the bacterial community structure and DOM molecules were characterized using Bray-Curtis dissimilarity indices. Community similarities were visualized through nonmetric multidimensional scaling (NMDS) plots and statistically analyzed by analysis of similarity (ANOSIM) and analysis of variance (ANOVA), using the software PAST [56]. For bacterial communities and FT-ICR-MS analysis, the relative abundances of each OTU and molecular mass were used to calculate pairwise similarities among samples using the Bray-Curtis coefficient. Principal component analysis (PCA) for bacterial community analysis was performed using the variance-covariance matrix of the normalized data. The loadings of OTUs with scores of ±0.05, which identifies absolute correlations between synthetic and original variables, were used to identify those variables mainly contributing to the variation in the data set [57]. In contrast to NMDS, PCA identifies only linear relationships and was therefore used only to assess OTU patterns in the dataset. However, a PCA identifies links between the principle components and OTUs. Since most (>90%) of the variance could be explained by the first three principle components, the PCA was statistically successful [57]. The ANOSIM for DOM compounds was performed by defining the different mesocosm set-ups as groups (cRW, cBS, RW, LYO, ULTRA) and applying pairwise comparisons using the Bray-Curtis dissimilarity index. Repeated measure analysis of variance (rmANOVA) in BP and TBN was always applied to identify significant differences between treatments for which we assumed normal distribution. For DOC, an ANOVA was used since only the relative changes between the beginning and end of the experiment were taken into account, because of the different starting conditions.

Bacterial community evenness, which considers the number of each species in an environment, was calculated based on Buzas and Gibson’s evenness, e^H′^/S (where H′ is the Shannon Weaver index and S is the species number), implemented in PAST.

## Results

### Decomposition of DOC and Changes in Bacterial Activity

**Figure 3:**
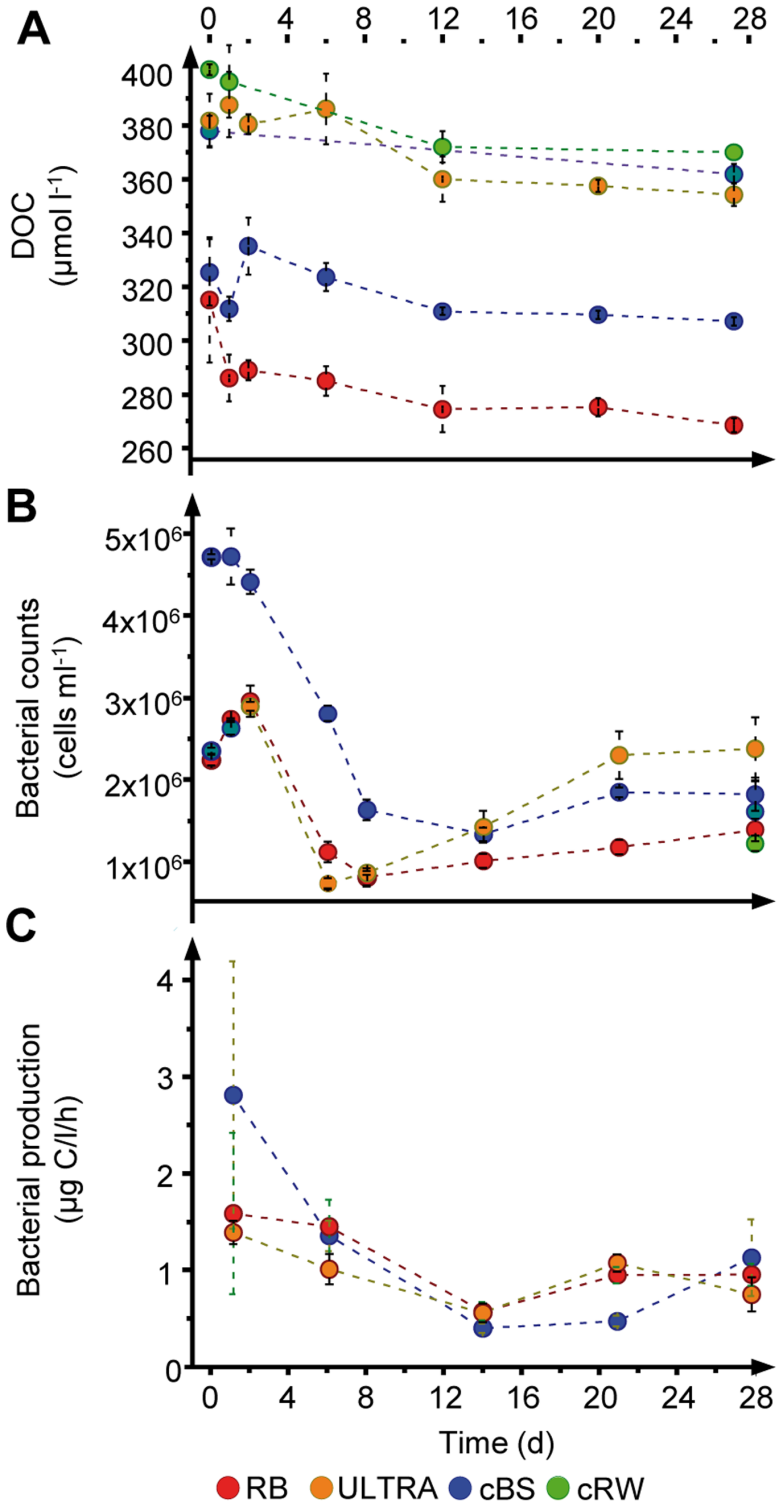
Bacterial and dissolved organic carbon dynamics in the experiment. Changes in (A) the dissolved organic carbon (DOC) concentration, (B) bacterial abundance, and (C) bacterial production in the mesocosm experiments from day 0 to day 28. The means and standard deviations of three independent mesocosms are shown (same abbreviations as in Fig. 1).

In the mesocosms, the DOC concentration over the 28-day incubation period decreased significantly [cBS 6% (±3), cRW 7% (±1), ULTRA 7% (±3), LYO 4% (±1), RB 16% (±6)] (Fig. 3) but without statistically significant differences between the control and the manipulated conditions (ANOVA, p>0.05, Table S4). Most of the DOC removal took place within the first 12 days of the experiment (Fig. 3A). Total prokaryotic cell numbers (TCN) decreased strongly after day 2 in all treatments and only recovered slightly towards the end of the experiment (Fig. 3B). At least for the measured time points in the RB, cBS, and ULTRA mesocosms, a comparable pattern in the time course of BP was apparent. Thus, BP was highest during the first few days of the experiment, although with large variability among the replicates (Fig. 3C). Thereafter, the variability decreased such that BP was lowest at day 14 of the experiment before slightly increasing towards the end of the experiment (Fig. 3C). However, there was no significant difference in BP (rmANOVA, p>0.05) between the RB, cBS, and ULTRA mesocosms. The mesocosms cRW and LYO had too few sampling points to be included in the statistical analysis. Bacterial carbon demand was highest on day 6, ranging from 10 (±4) to 20 (±9) μg C L^−1^ h^−1^ (Table S5), and bacterial growth efficiency was lowest at the end of the experiment (Table S5). For HNAN, the initial 2.6–6.3 × 10^3^ cells ml^−1^ recorded on the first day of the experiment decreased to 0.8–2.6 × 10^3^ and 0.8–3.1 × 10^3^ cells ml^−1^ on days 2 and 8, respectively (Table S6). HNAN counts did not change significantly between these two days (ANOVA, p>0.05). Inorganic nitrogen was not limiting throughout the experiment (Figure S3); however, the measured phosphate concentrations in all mesocosms to which artificial seawater had been added were lower (0.03 μM) than expected from the nutrient addition (1 μM PO_4_). We assume that this was caused by the precipitation of phosphate with the increased iron concentration in the artificial sea water. There was, however, no evidence that the lower phosphate concentration had an effect on bacterial activity in the experiment.

### Changes in Molecular DOM Composition

**Figure 4:**
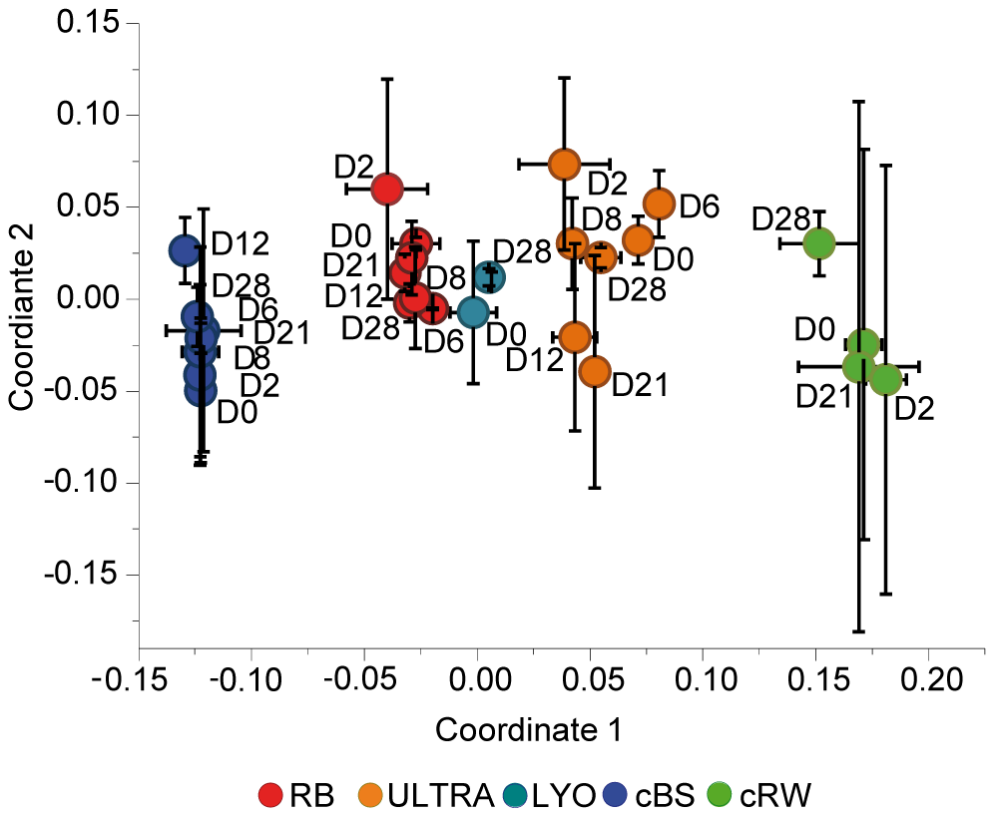
The dynamics of dissolved organic matter. Non -metric multidimensional scaling plot of the signal intensities of >4600 molecules measured by Fourier-transform ion cyclotron resonance mass spectrometry (D = day). The means and standard deviations of three independent mesocosms are shown (same abbreviations as in Fig. 1).

More than 4,600 different molecular formulas of DOM compounds, detected in >80% of the experimental samples, were considered for the statistical analysis (Table S3). NMDS plotting and Bray-Curtis dissimilarity analysis of the captured DOM molecules distinguished in the first coordinate the different mesocosms (cBS, cRW, RW, ULTRA, LYO) of the experiment (Fig. 4) and clearly showed that the greatest difference was between the Baltic Sea mesocosm (cBS), representing the most “marine” conditions in our experiment, and the freshwater control (cRW) mesocosm, containing the highest proportion of terrigenous DOM. The results from the mesocosms in which Baltic Sea water and a tDOM source were mixed were clearly separated from each other and located between those of the two control treatments (Fig. 4). The alternative noise reduction approach where only those compounds were considered that were present in all triplicate samples showed a similar trend (Figure S1). Using a relative proportion of 43% tDOM as the basis for the central Baltic Sea water [58] and 100% tDOM for Kalix River water, we estimated the relative tDOM concentrations in all treatments based on the measured DOC concentrations. This resulted in a tDOM amount of 81% for the 0.2-μm-filtered Kalix River water mixed with Baltic Sea water (RB) and 84% for the mesocosms containing ultrafiltrated Kalix River water+Baltic Sea water (ULTRA) and lyophilized Kalix River water+Baltic Sea water (LYO).

The relative contribution of tDOM in each treatment is indicated in the first coordinate of the NMDS (Fig. 4), which separates the different experimental treatments. The second coordinate shows a random distribution of the sampling times and other parameters. Additional statistical analyses (ANOSIM, Table S7) supported this result and suggested that the investigated DOM molecules varied only slightly between time points, without a consistent temporal trend in the experiments in terms of degradation or the generation of new molecules. Some change in the molecular composition over time was indicated in single mesocosms (Figure S4), but statistically significant changes were obscured by the differences in the DOM compositions between replicate mesocosms. This is supported by an analysis of a randomly generated dataset which yielded a similar number of molecules that changed significantly between day 0 and 28 (Figure S4).

### Bacterial Community Analysis

**Figure 5:**
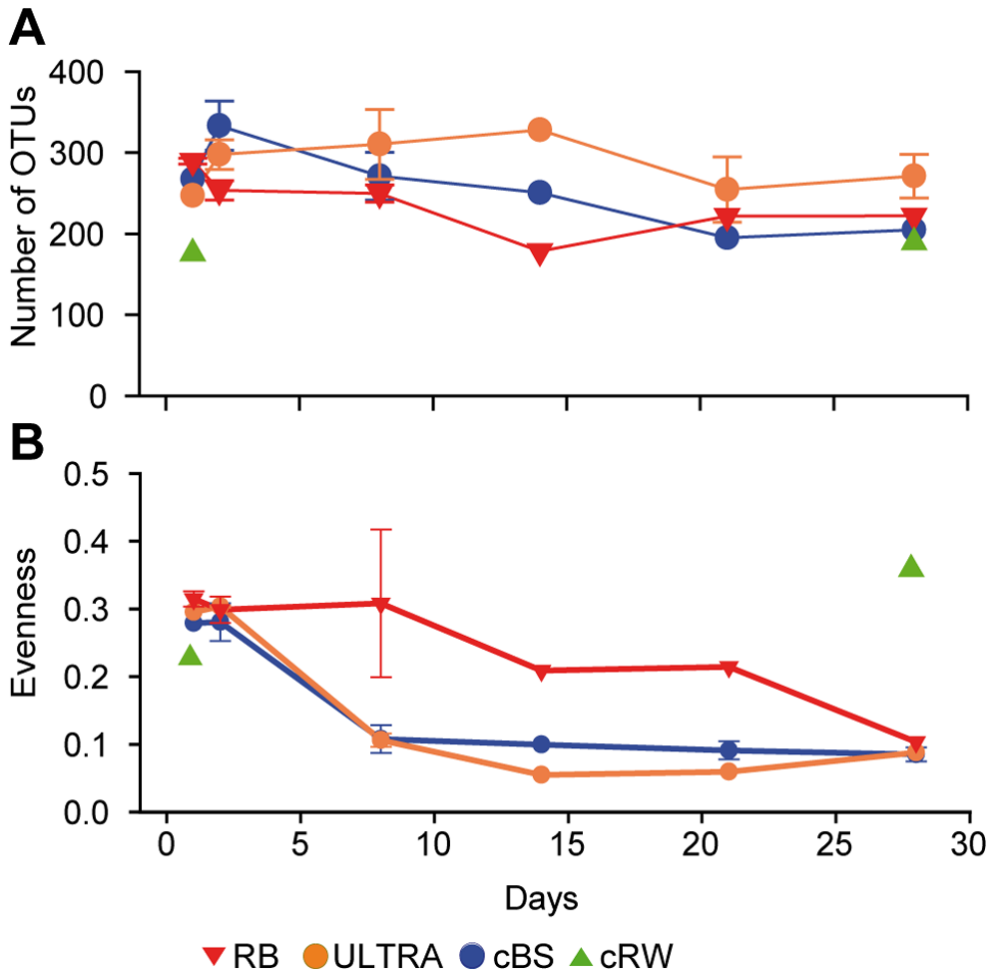
Bacterial diversity. Changes in (A) the number of OTUs and (B) bacterial evenness in mesocosms incubated for 28 days. The means and standard deviations of three independent mesocosms are shown (same abbreviations as in Fig. 1).

The total number of OTUs investigated during the 28-day incubations decreased in both the cBS and ULTRA mesocosms [from 267 (±52) to 204 (±28) and from 290 (±3) to 222 (±55), respectively] but increased in the RB mesocosms [from 247 (±16) to 271 (±27)] (Fig. 5A). However, there was no significant difference in OTU numbers (two-way ANOSIM, p>0.05) between treatments or days. The evenness of the bacterial community was dramatically lower after day 2 in the mesohaline mesocosms (Fig. 5B), independent of the treatment (two-way ANOSIM, p>0.05). This decrease paralleled that in cell abundance. Unlike the other treatments, evenness in the freshwater control mesocosm (cRW) slightly increased between the beginning and end of the experiment (Fig. 5B).

**Figure 6:**
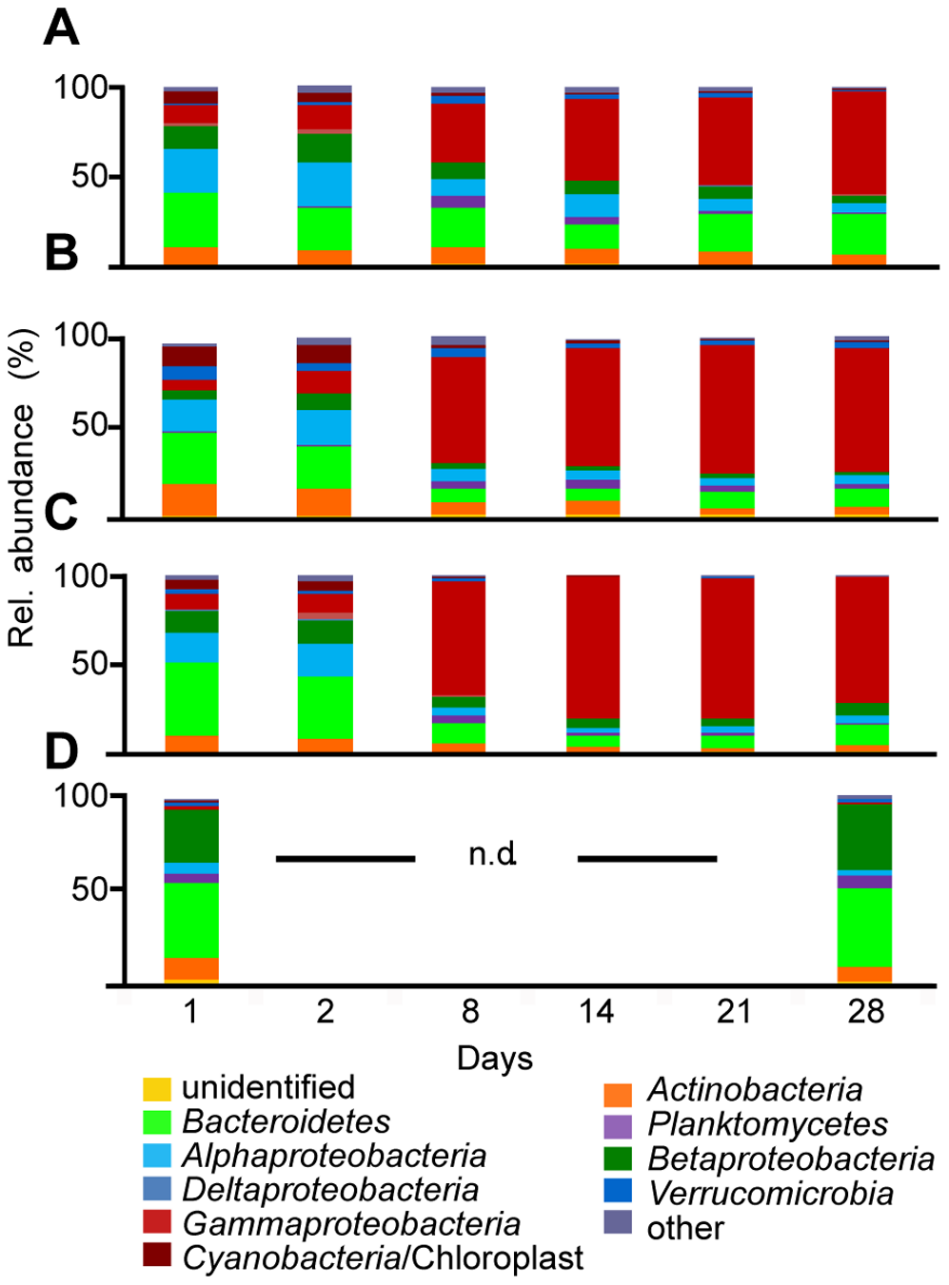
Bacterial community composition. Phylogenetic composition of the major bacterial phyla (and proteobacterial subclasses) in the mesocosms. The relative abundance of each phylum is expressed as the mean percent of total sequences obtained in three independent mesocosms. (A) RB, (B) ULTRA, (C) cBS, (D) cRW (nd =  not determined; same abbreviations as in Fig. 1).

To understand the changes in bacterial community structure that occurred during the 28-day incubation, we classified the OTUs based on their phylogenetic affiliations and determined their relative proportions of the total pyrosequencing reads. On days 1 and 2 of the experiment, the mesohaline mesocosms (cBS, RB, ULTRA, and LYO) contained a diverse bacterial community that included the phyla *Actinobacteria*, *Proteobacteria*, *Bacteroidetes*, *Cyanobacteria*, *Planctomycetes*, and *Verrucomicrobia* (Fig. 6 A–C). Between days 2 and 8, the bacterial community composition (BCC) of these mesocosms changed dramatically, towards one dominated by *Gammaproteobacteria*, which comprised 32–64% of all pyrosequencing reads. The most abundant representative was an OTU related to *Paraperlucidibaca baekdonensis* (Figure S5, accession no. GU731671), which started to dominate (11–44%) the BCC on day 8 (Figure S6). Another *Gammaproteobacterium*, related to *Zhongshania antarctica* (Figure S5, accession no. FJ889619), was abundant in the cBS mesocosm after 14 days [23% (±7)]. The relative abundance of this OTU increased in the RB and ULTRA treatments as well but with a lower relative intensity [RB 3% (±2); ULTRA 7% (±1)]. Few of the other OTUs from the initial Baltic Sea community increased in abundance (Figure S7) and their influences on the PCA were accordingly minor (Table S8).

In contrast to these results, a diverse microbial community occurred in the river water control mesocosms, in which *Actinobacteria*, *Alphaproteobacteria*, *Betaproteobacteria*, *Bacteroidetes*, and *Verrucomicrobia* were detected. This BCC remained relatively constant during the 28-day incubation (Fig. 6D). A change in the BCC of the freshwater mesocosm was primarily reflected in the second principle component of the PCA (Figure S8) and could largely be attributed to an OTU assigned to a flavobacterial phylotype. The relative abundance of this OTU, related to sequences identified in phytoplankton blooms in Lake Zürich ([59], Figure S5, accession no. HE574390), decreased from almost 25% to 3% in the cRW mesocosms (Figure S7). Moreover, unlike in the mesohaline mesocosms, in the freshwater control mesocosms a diverse microbial community was maintained throughout the experiment.

**Figure 7:**
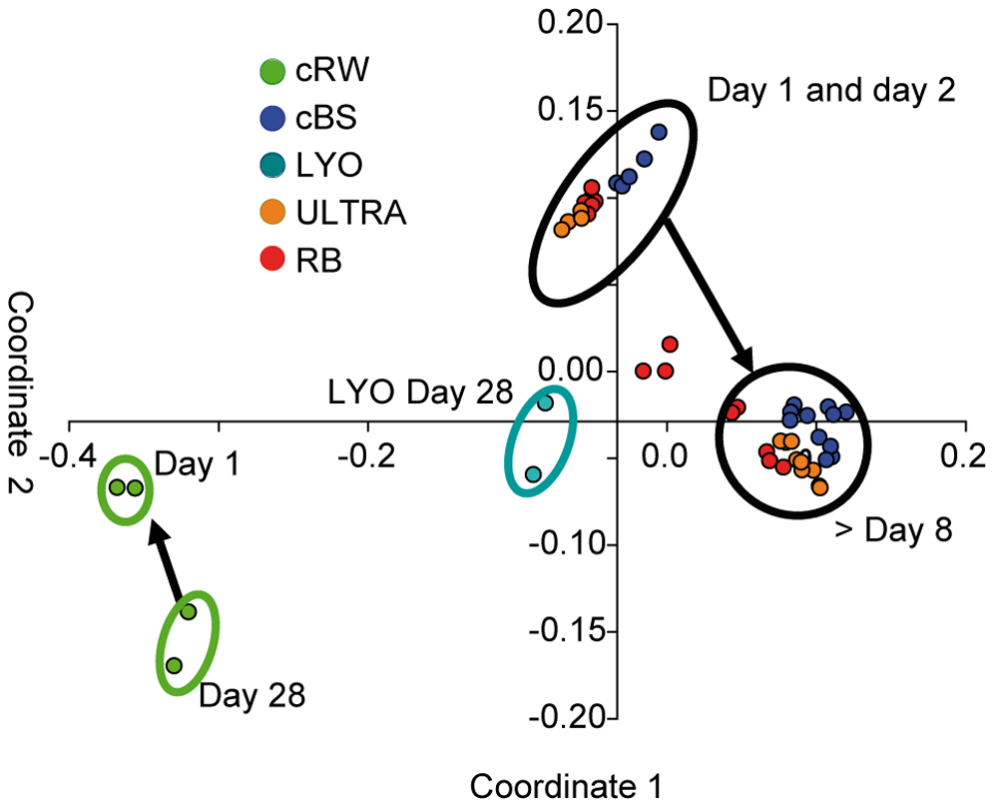
Non-metric multidimensional scaling plot of the bacterial community composition based on operational taxonomic units. Orange lines mark the change in bacterial community composition of the RB, ULTRA, and cBS mesocosms, light green lines the freshwater control mesocosm. Dark green circle represents the bacterial community of LYO after 28(same abbreviations as in Fig. 1).

In concordance with the results of the PCA (Figure S8), the difference between the freshwater and mesohaline mesocosms was supported by the NMDS diagram (Fig. 7), the first coordinate of which showed a clear separation between mesohaline and freshwater bacterial communities. On the second coordinate, a temporal shift in the bacterial community of the mesohaline mesocosms was evident between days 1 and 2 and after day 8.

## Discussion

### Uncoupling of Bacterial and tDOM Dynamics

The aim of this study was to assess the biodegradability of tDOM by mesohaline bacterial communities as well as the interactions between DOM components and bacterioplankton composition. Ultrahigh-resolution mass spectrometry showed that tDOM addition significantly altered the DOM composition in the mesocosms, with differences found also in terms of the added tDOM preparations (Fig. 4). However, comparisons of bacterial community development, activities, and DOC removal between mesocosms with tDOM additions and those of the unamended controls revealed that, overall, tDOM addition had only a minor effect on the dynamics of bacteria and DOM (Figs. 3, 4, 6). This unexpected result suggested that the bacterial communities in the mesocosms were not capable of utilizing the added tDOM and that its bioavailable fraction was relatively small, at least under the conditions of our experiment.

The significant bacterial activity and the pronounced bacterial succession, which were similar in the unamended controls and tDOM-modified mesocosms, indicated that bacterial dynamics were largely uncoupled from the tDOM additions but fueled by the DOM already present in Baltic Sea water. Most of the DOC decomposition took place within the first few days of the experiment, most likely because of the degradation of an uncharacterized labile DOM pool (Fig. 3). After 12 days, the majority of the DOC, including the added tDOM, probably consisted of semi-labile and recalcitrant components with turnover times exceeding the time span of our experiment (>1 month). The poor bioavailability of DOM was also evidenced by the low efficiency of bacterial growth at the end of the experiment (Table S4). Prior to the exhaustion of the labile DOM, there were strong declines in bacterial abundance, production (Fig. 3), and evenness (Fig. 5) that may have been related to the depletion of labile organic substrates and to continued grazing by small protists, judging from the relatively high HNAN numbers (Table S6).

FT-ICR-MS is a highly advanced method for obtaining DOM fingerprints and characterizing DOM components [19], [60] but it does not cover the entire DOM spectrum, including some groups of molecules that potentially fueled bacterial activity in the mesocosms. For example, sample preparation for FT-ICR-MS (SPE-DOM) excludes both small monomeric compounds and colloidal matter [39]. Both size classes contain potentially labile DOM fractions. The ionization mode in FT-ICR-MS and the resulting ionization efficiencies have been shown to additionally influence the spectrum and resolution of the assessed DOM compounds [60]. Negative ion mode in electrospray ionization, used in this study, accurately detects acidic functional groups but may exclude some nitrogen-containing compounds [61]. This would explain the observed patterns of bacterial production and DOC decomposition despite the lack of a significant temporal trend in bulk DOM. A decrease in DOC concentration can also be caused by physical flocculation, for example, during the artificial increase in salinity in the tDOM-amended mesocosms [62], thereby resulting in an overestimation of microbial DOC utilization. However, since the levels of bacterial activity and DOC utilization in the tDOM-amended and control mesocosms were comparable, we consider this mechanism to have been of minor importance in our experiment.

In accordance with our results, previous studies examining subarctic tDOM utilization in incubation experiments reported little or no DOC decomposition [14], [63]. The high abundance of lignin and other vascular-plant-derived components in tDOM was interpreted as contributing to its stability in the incubation experiments [14], [64]. This observation contrasts with those from studies showing tDOM removal during estuarine mixing under the oligohaline conditions of the Northern Baltic Sea [58], [65]–[67]. The underlying reasons for these contrasting results may be strong seasonal [67] and regional [68] differences in biodegradability. Moreover, photo-degradation [69] or flocculation because of increased salinity [62] could also account for tDOM removal in the Northern Baltic Sea.

### Bacterial Community Dynamics

In the mesohaline mesocosms, the BCC underwent very large temporal shifts, with a similar succession in all treatments, independent of tDOM addition (Fig. 6). The change in the BCC was accompanied by a significant decrease in evenness (Fig. 5), which reflected the dominance of one gammaproteobacterial OTU affiliated with *Paraperlucidibaca*. This OTU was not detected in our previous larger-scale survey of BCC in the Baltic Sea [48]. *Paraperlucidibaca* phylotypes were reported in a study of psychrotolerant semi-labile crude-oil degradation in Antarctic waters [70]. The dominance of this phylotype during the later stage of our experiments suggests that this bacterium is capable of using semi-labile DOM. This conclusion is supported by the low growth efficiency measured towards the end of the experiment. It may be that longer incubation times are necessary before measurable degradation of semi-labile DOM can be recognized.

A shift towards the dominance of a *Paraperlucidibaca* phylotype was not observed in the freshwater control mesocosms (cRW), although this bacterium should be able to grow in freshwater conditions [71]. However, the initial BCC in cRW differed from that of the mesohaline mesocosms (Fig. 6) and was not subject to an increase in salinity, suggesting that the initial bacterial community also had a strong impact on bacterial succession. Interestingly, in the cRW mesocosm a DOC utilization rate comparable to that of the other mesocosms was measured, which in this case should have involved mainly tDOM. This suggests that under freshwater conditions a fraction of riverine tDOM is bioavailable. However, none of the bacteria present in the freshwater mesocosm were stimulated in the tDOM-amended mesohaline mesocosms; thus, either they were absent from the mesohaline inoculum or the salinity was high enough to suppress their development. An impact of salinity on bacterial dynamics seems more likely since saline conditions were shown in previous studies to determine BCC [72], [73].

### Limitations of Incubation Experiments in Examining tDOM Decomposition

Studies on the biodegradation of semi-labile tDOM are complicated by the need for relatively long (weeks to months) incubation times to detect significant decomposition rates (*e.g.*, [74]) but this in itself triggers changes in microbial community composition and activities. The confinement of marine or freshwater samples was previously shown to result in shifts in prokaryotic community structure [75]–[77] and to stimulate bacterial activity and the development of bacterivorous protists, even without substrate amendment [78], [79]. Although changes in community structure do not always imply changes in general microbial activity [75], it is hardly possible to judge whether the processes observed during the incubations mimic those that occur *in situ*.

Similarly, in our experiment the abundances of bacterial groups typical of the mesohaline part of the Baltic Sea, such as the alphaproteobacterial SAR11 [48] and the verrucomicrobial “Spartobacteria baltica” [80], declined during the incubation (Fig. 6). The *Paraperlucidibaca* phylotype, which became dominant during the incubation, was not detected at the beginning of the experiment, nor does it occur in the Baltic Sea under summer conditions [48]. A shift towards a gammaproteobacterial-dominated bacterial community in seawater incubation experiments has been frequently reported [24], [81]–[83] and was also observed in a study using Baltic Sea water [84]. This effect has been recognized not only in batch mesocosm experiments but also in continuous cultures [83], [85], [86], in macrocosms [87], and even *in situ*, during the large oil spill in the Gulf of Mexico in 2010 [88]. Therefore, the change in BCC and the decline in bacterial abundance and growth efficiency during the incubations together point to an exhaustion of labile organic substrates and the ability of the *Paraperlucidibaca* phylotype to degrade semi-labile DOM under mesohaline conditions. However, it is also possible that this phylotype is resistant to grazing or produces antibiotics and therefore ultimately dominates in the experiment.

Remarkably, the BCC in the freshwater control mesocosm (cRW) did not undergo the dramatic shift seen in the mesohaline mesocosm (cBS) nor did a gammaproteobacterial phylotype become dominant (Fig. 6). This finding indicates that sample treatment can be ruled out as a major factor influencing BCC development and it underlines the importance of the original composition of the prokaryotic community. Grazing by small bacterivores can also strongly shape prokaryotic succession, possibly shifting it towards less edible species [89]. In addition, BCC may be altered by viral lysis [90], [91], but whether this occurred in our experiments was not determined. Grazing and viral lysis can add recalcitrant carbon compounds to the DOM pool [92] and thus modify both the quantity and the composition of the DOM.

Among the factors influencing bacterial community and DOM composition in our experiments, the contribution of tDOM was judged to be smaller than the roles of grazers and the composition of the bacterial inoculum. Especially at low rates of biodegradation because of the refractory nature of the supplied DOM, effects attributable to DOM addition will be overshadowed by a strong confinement-dependent succession in microbial structure. Our observations underline the continued challenge of interpreting long-term incubation studies, which inevitably involve major changes in microbial communities and deviations from *in situ* conditions.

### Conclusion

Our results showed the similar development of control and manipulated mesocosms with respect to bacterial activity, DOC removal, and, for the mesohaline conditions, also bacterial community composition. Under the applied experimental conditions, the addition of tDOM had no measurable effect on any of these parameters, indicative of the semi-labile character of the added subarctic tDOM. This result is independent of the chemical modification of tDOM by the preparation and instead suggests that the major part of tDOM is recalcitrant to bacterial degradation. Indeed, the driving forces underlying the bacterial dynamics determined in our experiments were more likely attributable to the origin of the bacterial community (linked to salinity) as well as to enclosure and grazing. Our findings point out the difficulties encountered in attempts to analyze small effects caused by tDOM addition in incubation experiments such as those described herein. In addition, the natural conditions in an estuary, which include variations in salinity and nutrients, solar radiation, and DOM production by algal exudation, could not be accounted for in this mesocosm experiment and thus may have led to an underestimation of natural tDOM degradation. Nonetheless, according to our results, the semi-labile character of tDOM, in combination with the low temperature of the water in subarctic estuaries, should limit bacterial tDOM decomposition in these environments. The contributions of other processes to tDOM removal, including physical removal by flocculation or photo-oxidation and subsequent bacterial utilization, may be more important.

## Supporting Information

Figure S1 **Non -metric multidimensional scaling (NMDS) plot of the Fourier-transform ion cyclotron resonance mass spectrometry signal intensities of detected molecular masses in triplicate mesocosms of one treatment.** For abbreviation of the treatments see Fig 1. Masses detected only in one or two of the three parallel experiments were excluded (D = day). Shown are averages with standard deviation of three independent mesocosms. The Coordinate 1 does not distribute the sample along a time axis or any other known parameter. In contrast to this, the coordinate 2 separates between the different treatments. Compared to Fig. 2, the samples are more randomly distributed in the NMDS, because the less restrict filtering criteria introduced more analytical noise.
(PDF)

Figure S2 **Changed molecular intensities between day 0 and day 28 of selected samples.** Van Krevelen plots of molecular formulae whose Fourier-transform ion cyclotron resonance mass spectrometry (FT-ICR-MS) signal intensities significantly varied between day 0 and day 28 (paired Student’s t-test, p<0.05), exemplarily shown for the control experiments (cBS, A) and Kalix river manipulation (RB, B). If the FT-ICR-MS signal intensities are randomly redistributed among the ∼4000 molecular formulae, a similar number of significant differences is found (panel C). This example illustrates that any trend observed in the course of experiments is undistinguishable from a randomly generated data set, *i.e.*, not significant.
(PDF)

Figure S3 **Nutrients measured during the experiment.** Shown is the average concentration of three independent replicated mesocosms: (A) NO_2_, (B) PO_4_, (C) NO_3_, (D) SiO_4_. For abbreviation of the treatments see Fig 1.
(PDF)

Figure S4 **Van Krevlen Plots of all triplicates of the cBS and RB samples at day 0 and 28.** For abbreviations of the treatments see Fig. 1. Each plot contains about 4000 molecules whereas the relative molecular intensities were interpolated (weighted average gridding). Images were created with Ocean Data View (Schlitzer, R., Ocean Data View, http://odv.awi.de, 2013).
(PDF)

Figure S5 **Phylogenetic tree of the operational taxonomic units with high loadings in the Principle Component Analysis.** Full length 16S rRNA sequences (>1000 bp) were used to calculate a core tree based on related environmental and reference sequences, using maximum-likelihood analysis (PhyML). Short sequences from this study (300 bp), were added without changing the global tree topology using the ARB parsimony tool. Numbers indicate the link to Figure S6.
(PDF)

Figure S6 **Relative proportion of the** ***Paraperlucidibaca*** **assigned pyrosequencing reads in the mesocosms.** Shown is the relative average abundance of three replicated mesocosms. For abbreviation of the treatments see Fig 1.
(PDF)

Figure S7 **Heatmap of the relative proportion of pyrosequencing reads assigned to a operational taxonomic unit (OTUs).** The heatmap shows OTUs of replicated mesocosms with high loadings (±0.05) in the Principle component analysis from the mesocosm experiments. For abbreviation of the treatments see Fig 1.
(PDF)

Figure S8 **Principle component analysis (PCA) of changes in the operational taxonomic units (OTU) composition during the experiment.** The PCA is basis for the OTU loadings in Figure S7. Eigenvalue PC1 = 80.6%; PC2 = 6.6%; PC3 = 5.0. For abbreviation of the treatments see Fig 1.
(PDF)

Table S1 **Chemical composition of Artificial Sea Water.** Solutions (A), (B) and (C) were separately prepared with ultrapure water.
(PDF)

Table S2 **Experimental set up and conditions of the mesocosms.** Abbreviations for treatments as in Fig. 1, solutions A, B, C see Table S1.
(PDF)

Table S3 **List of assigned dissolved organic matter compounds.**
(TXT)

Table S4 **Statistical analysis of the dissolved organic carbon (DOC) decomposition in the experiments.** (A) DOC decomposition in the mesocosms (same abbreviations as in Fig. 1). (B) Based on the DOC reduction, no significant difference in a one-way ANOVA test can be determined.
(PDF)

Table S5 **Bacterial activity and bacterial growth efficiency.** Bacterial activity estimated by [^3^H]-thymidine incorporation and oxygen consumption measured by a luminescence-based optical sensor (nd =  not determined; same abbreviations as in Fig. 1).
(PDF)

Table S6 **Nanoflagellate counts (ml^−1^) in the mesocosms.** For abbreviations see Fig. 1.
(PDF)

Table S7 **ANOSIM test of the dissolved organic matter composition.** ANOSIM test of all day 0 and day 28 samples from the treatments based on Bray Curtis dissimilarity, calculated with PAST, (A) represent the R- values and (B) the uncorrected p-values.
(PDF)

Table S8 **Loadings of a PCA analysis of the operational taxonomic units (OTUs) in the experiments.** Loadings of a PCA analysis indicating OTUs contributing >0.05 or <0.05 to a principle component.
(PDF)
