# Supplementary material for: Uncoupling of Bacterial and Terrigenous Dissolved Organic Matter Dynamics in Decomposition Experiments
Source: PLoS One. 2014 Apr 9;9(4):e93945. doi: 10.1371/journal.pone.0093945 (PMC3981725; doi:10.1371/journal.pone.0093945)
Supplement: Figure S1 — Non -metric multidimensional scaling (NMDS) plot of the Fourier-transform ion cyclotron resonance mass spectrometry signal intensities of detected molecular masses in triplicate mesocosms of one treatment. For abbreviation of the treatments see Fig 1. Masses detected only in one or two of the three parallel experiments were excluded (D = day). Shown are averages with standard deviation of three independent mesocosms. The Coordinate 1 does not distribute the sample along a time axis or any other known parameter. In contrast to this, the coordinate 2 separates between the different treatments. Compared to Fig. 2, the samples are more randomly distributed in the NMDS, because the less restrict filtering criteria introduced more analytical noise. (PDF) [file pone.0093945.s001.pdf]

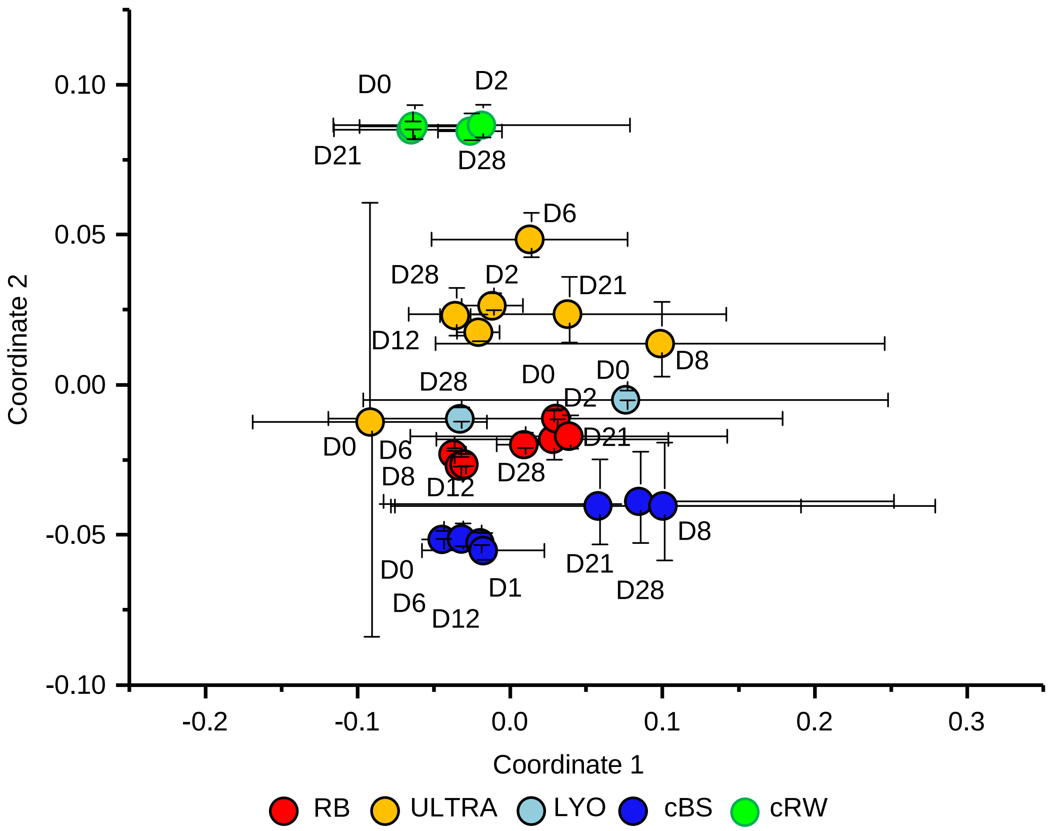

Figure S1. Non -metric multidimensional scaling (NMDS) plot of the Fourier-transform ion cyclotron resonance mass spectrometry signal intensities of detected molecular masses in triplicate mesocosms of one treatment. For abbreviation of the treatments see Fig 1. Masses detected only in one or two of the three parallel experiments were excluded (D = day). Shown are averages with standard deviation of three independent mesocosms. The Coordinate 1 does not distribute the sample along a time axis or any other known parameter. In contrast to this, the coordinate 2 separates between the different treatments. Compared to Fig. 2, the samples are more randomly distributed in the NMDS, because the less restrict filtering criteria introduced more analytical noise.
