# Supplementary material for: Uncoupling of Bacterial and Terrigenous Dissolved Organic Matter Dynamics in Decomposition Experiments
Source: PLoS One. 2014 Apr 9;9(4):e93945. doi: 10.1371/journal.pone.0093945 (PMC3981725; doi:10.1371/journal.pone.0093945)
Supplement: Figure S2 — Changed molecular intensities between day 0 and day 28 of selected samples. Van Krevelen plots of molecular formulae whose Fourier-transform ion cyclotron resonance mass spectrometry (FT-ICR-MS) signal intensities significantly varied between day 0 and day 28 (paired Student’s t-test, p<0.05), exemplarily shown for the control experiments (cBS, A) and Kalix river manipulation (RB, B). If the FT-ICR-MS signal intensities are randomly redistributed among the ∼4000 molecular formulae, a similar number of significant differences is found (panel C). This example illustrates that any trend observed in the course of experiments is undistinguishable from a randomly generated data set, i.e., not significant. (PDF) [file pone.0093945.s002.pdf]

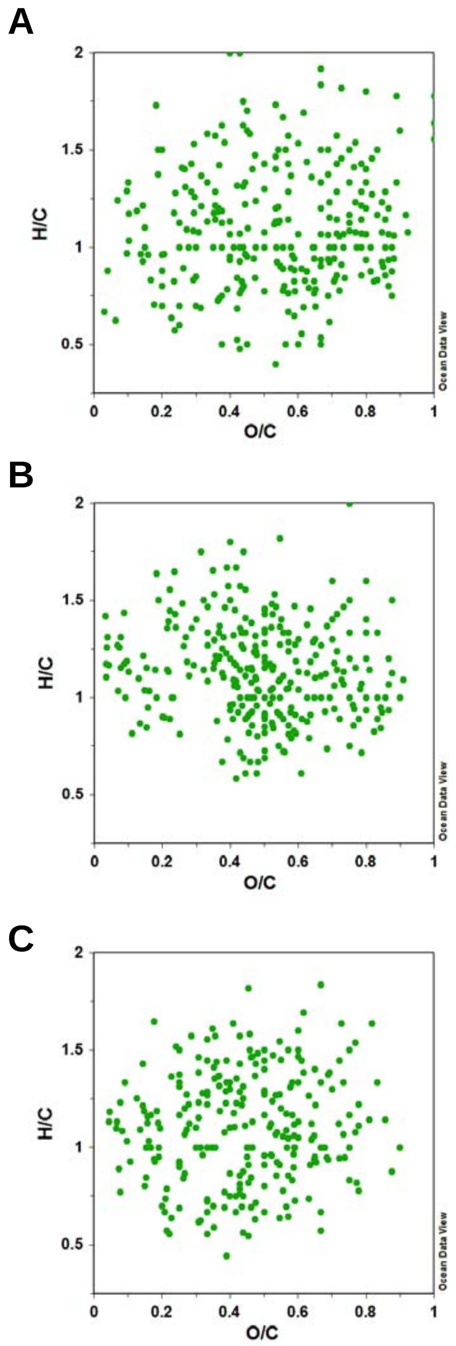

Figure S2. Changed molecular intensities between day 0 and day 28 of selected samples. Van Krevelen plots of molecular formulae whose Fourier-transform ion cyclotron resonance mass spectrometry (FT-ICR-MS) signal intensities significantly varied between day 0 and day 28 (paired Student's t-test, $p<0.05$), exemplarily shown for the control experiments (cBS, A) and Kalix river manipulation (RB, B). If the FT-ICR-MS signal intensities are randomly redistributed among the ~4000 molecular formulae, a similar number of significant differences is found (panel C). This example illustrates that any trend observed in the course of experiments is undistinguishable from a randomly generated data set, i.e., not significant.
