# Supplementary material for: Uncoupling of Bacterial and Terrigenous Dissolved Organic Matter Dynamics in Decomposition Experiments
Source: PLoS One. 2014 Apr 9;9(4):e93945. doi: 10.1371/journal.pone.0093945 (PMC3981725; doi:10.1371/journal.pone.0093945)
Supplement: Figure S3 — Nutrients measured during the experiment. Shown is the average concentration of three independent replicated mesocosms: (A) NO2, (B) PO4, (C) NO3, (D) SiO4. For abbreviation of the treatments see Fig 1. (PDF) [file pone.0093945.s003.pdf]

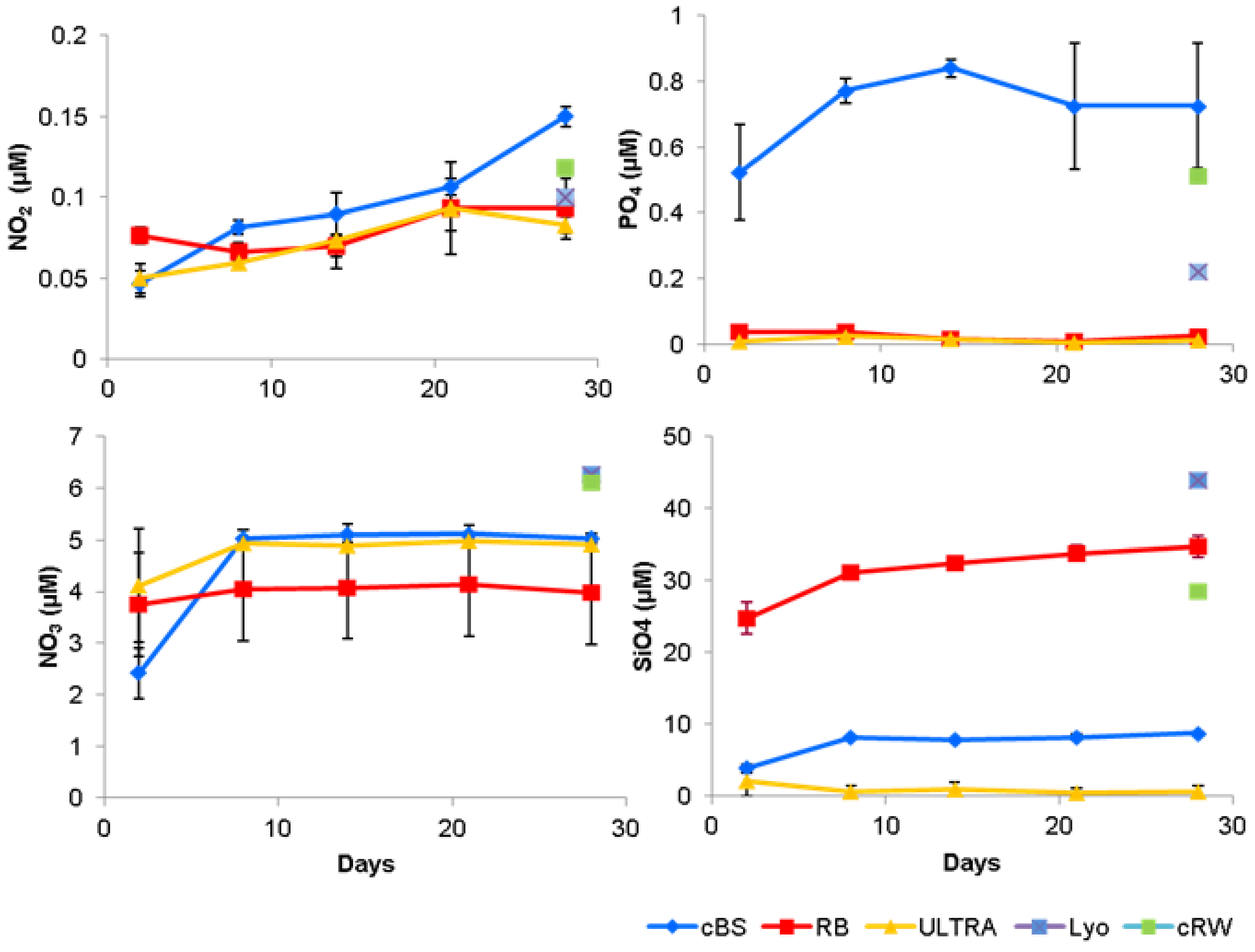

Figure S3. Nutrients measured during the experiment. Shown is the average concentration of three independent replicated mesocosms: (A) NO_2, (B) PO_4, (C) NO_3, (D) SiO_4. For abbreviation of the treatments see Fig 1.
