# Supplementary material for: Uncoupling of Bacterial and Terrigenous Dissolved Organic Matter Dynamics in Decomposition Experiments
Source: PLoS One. 2014 Apr 9;9(4):e93945. doi: 10.1371/journal.pone.0093945 (PMC3981725; doi:10.1371/journal.pone.0093945)
Supplement: Figure S4 — Van Krevlen Plots of all triplicates of the cBS and RB samples at day 0 and 28. For abbreviations of the treatments see Fig. 1. Each plot contains about 4000 molecules whereas the relative molecular intensities were interpolated (weighted average gridding). Images were created with Ocean Data View (Schlitzer, R., Ocean Data View, http://odv.awi.de, 2013). (PDF) [file pone.0093945.s004.pdf]

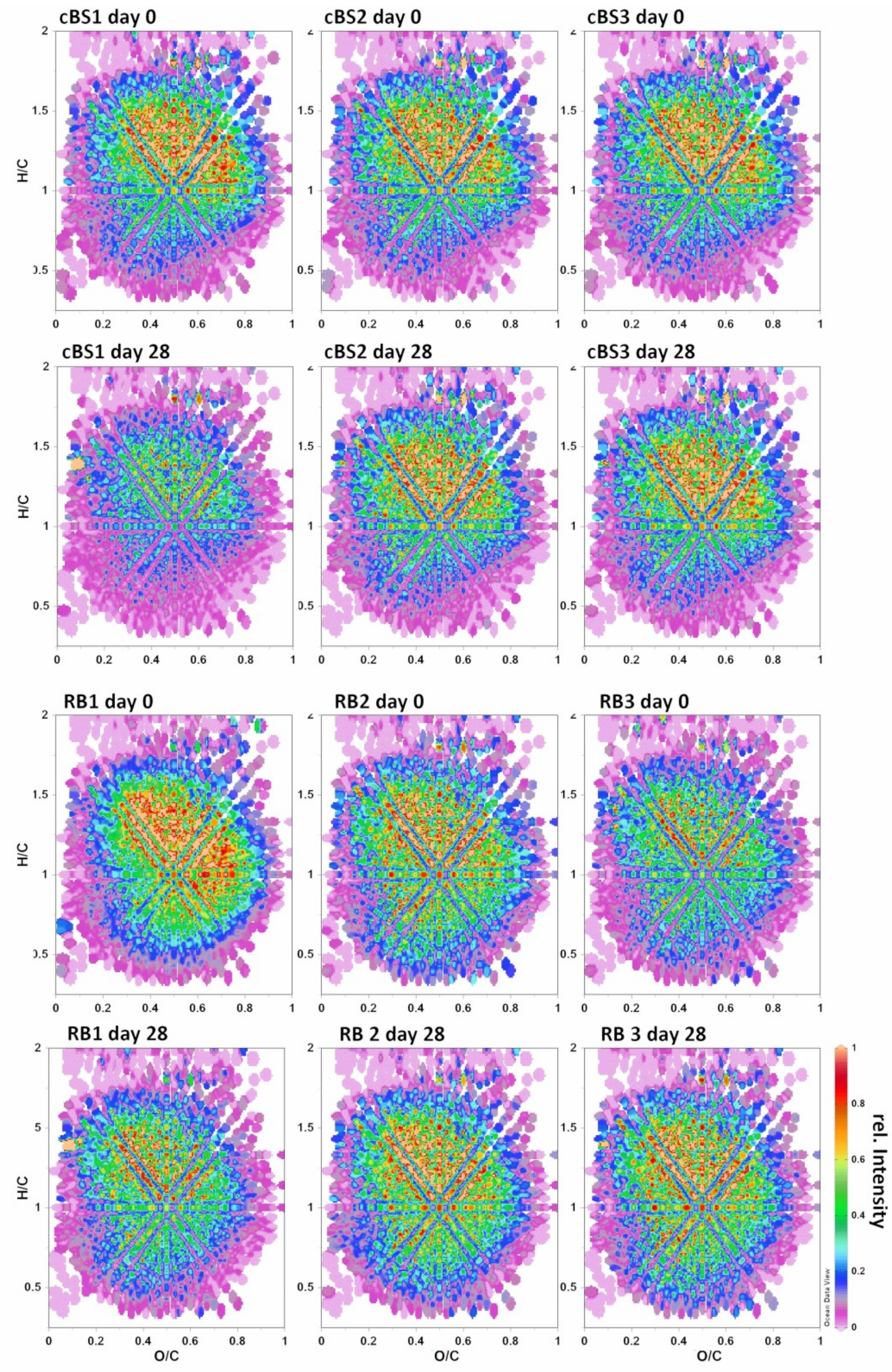

Figure S4. Van Krevlen Plots of all triplicates of the cBS and RB samples at day 0 and 28. For abbreviations of the treatments see Fig. 1. Each plot contains about 4000 molecules whereas the relative molecular intensities were interpolated (weighted average gridding). Images were created with Ocean Data View (Schlitzer, R., Ocean Data View, http://odv.awi.de, 2013).
