# Supplementary material for: Uncoupling of Bacterial and Terrigenous Dissolved Organic Matter Dynamics in Decomposition Experiments
Source: PLoS One. 2014 Apr 9;9(4):e93945. doi: 10.1371/journal.pone.0093945 (PMC3981725; doi:10.1371/journal.pone.0093945)
Supplement: Figure S5 — Phylogenetic tree of the operational taxonomic units with high loadings in the Principle Component Analysis. Full length 16S rRNA sequences (>1000 bp) were used to calculate a core tree based on related environmental and reference sequences, using maximum-likelihood analysis (PhyML). Short sequences from this study (300 bp), were added without changing the global tree topology using the ARB parsimony tool. Numbers indicate the link to Figure S6. (PDF) [file pone.0093945.s005.pdf]

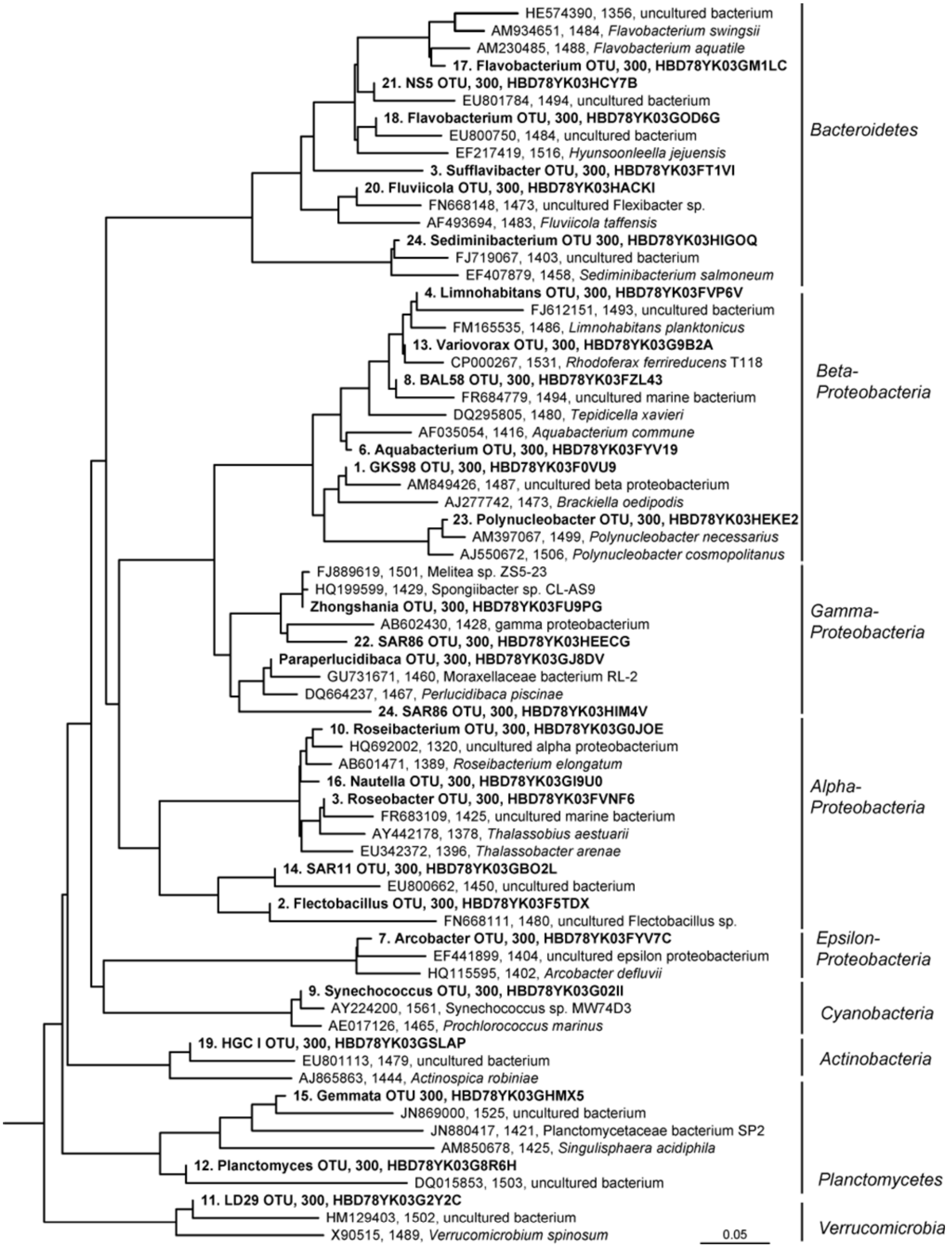

Figure S5. Phylogenetic tree of the operational taxonomic units with high loadings in the Principle Component Analysis. Full length 16S rRNA sequences (>1000 bp) were used to calculate a core tree based on related environmental and reference sequences, using maximum-likelihood analysis (PhyML). Short sequences from this study (300 bp), were added without changing the global tree topology using the ARB parsimony tool. Numbers indicate the link to Figure S6.
