# Supplementary material for: Uncoupling of Bacterial and Terrigenous Dissolved Organic Matter Dynamics in Decomposition Experiments
Source: PLoS One. 2014 Apr 9;9(4):e93945. doi: 10.1371/journal.pone.0093945 (PMC3981725; doi:10.1371/journal.pone.0093945)
Supplement: Figure S6 — Relative proportion of the Paraperlucidibaca assigned pyrosequencing reads in the mesocosms. Shown is the relative average abundance of three replicated mesocosms. For abbreviation of the treatments see Fig 1. (PDF) [file pone.0093945.s006.pdf]

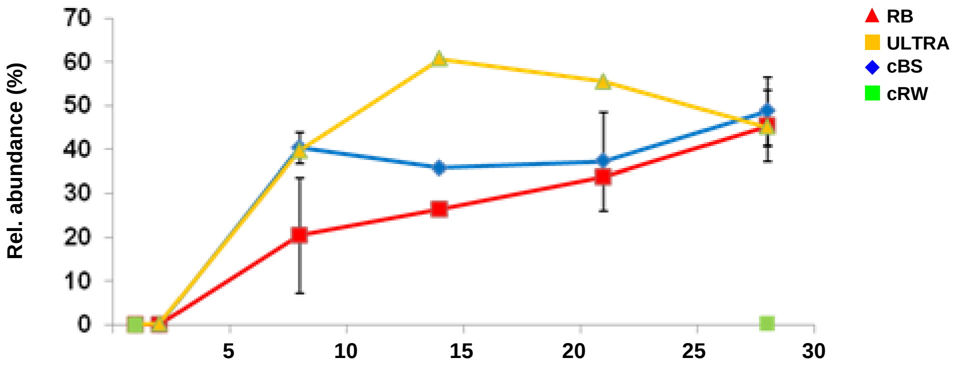

Figure S6. Relative proportion of the *Paraperlucidibaca* assigned pyrosequencing reads in the mesocosms. Shown is the relative average abundance of three replicated mesocosms. For abbreviation of the treatments see Fig 1.
