# Supplementary material for: Uncoupling of Bacterial and Terrigenous Dissolved Organic Matter Dynamics in Decomposition Experiments
Source: PLoS One. 2014 Apr 9;9(4):e93945. doi: 10.1371/journal.pone.0093945 (PMC3981725; doi:10.1371/journal.pone.0093945)
Supplement: Figure S7 — Heatmap of the relative proportion of pyrosequencing reads assigned to a operational taxonomic unit (OTUs). The heatmap shows OTUs of replicated mesocosms with high loadings (±0.05) in the Principle component analysis from the mesocosm experiments. For abbreviation of the treatments see Fig 1. (PDF) [file pone.0093945.s007.pdf]

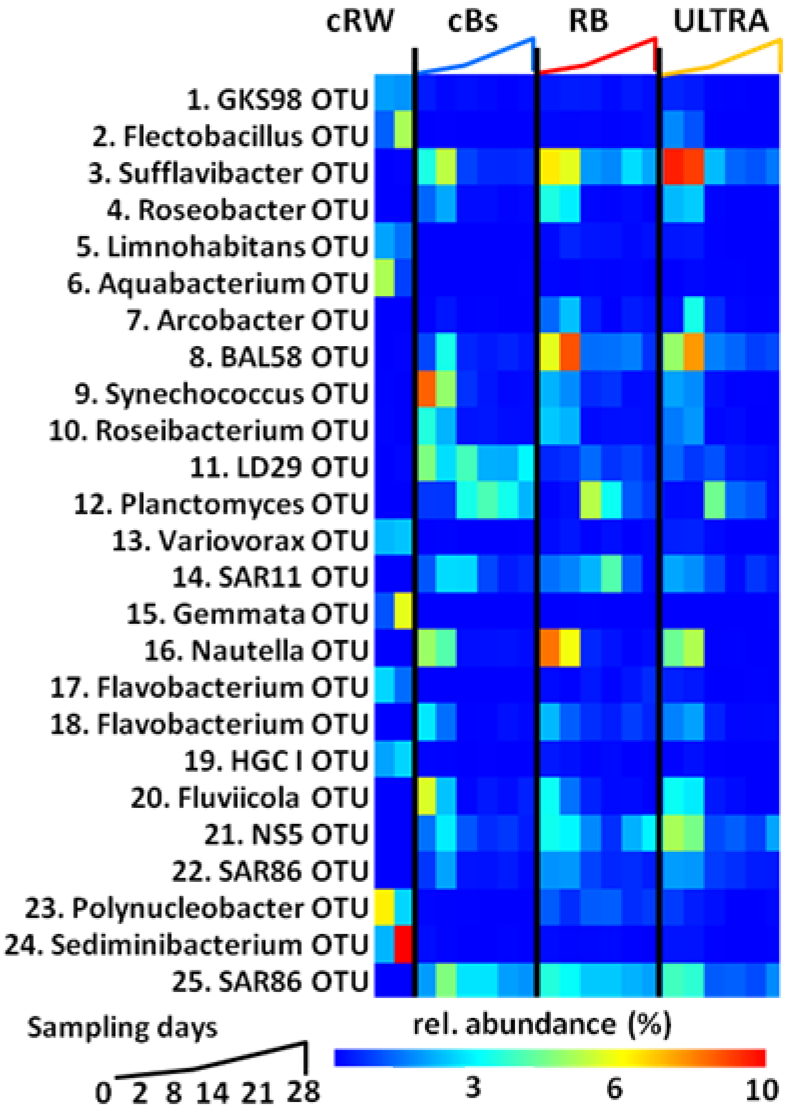

Figure S7. Heatmap of the relative proportion of pyrosequencing reads assigned to a operational taxonomic unit (OTUs). The heatmap shows OTUs of replicated mesocosms with high loadings (± 0.05) in the Principle component analysis from the mesocosm experiments. For abbreviation of the treatments see Fig 1.
