# Supplementary material for: Uncoupling of Bacterial and Terrigenous Dissolved Organic Matter Dynamics in Decomposition Experiments
Source: PLoS One. 2014 Apr 9;9(4):e93945. doi: 10.1371/journal.pone.0093945 (PMC3981725; doi:10.1371/journal.pone.0093945)
Supplement: Figure S8 — Principle component analysis (PCA) of changes in the operational taxonomic units (OTU) composition during the experiment. The PCA is basis for the OTU loadings in Figure S7. Eigenvalue PC1 = 80.6%; PC2 = 6.6%; PC3 = 5.0. For abbreviation of the treatments see Fig 1. (PDF) [file pone.0093945.s008.pdf]

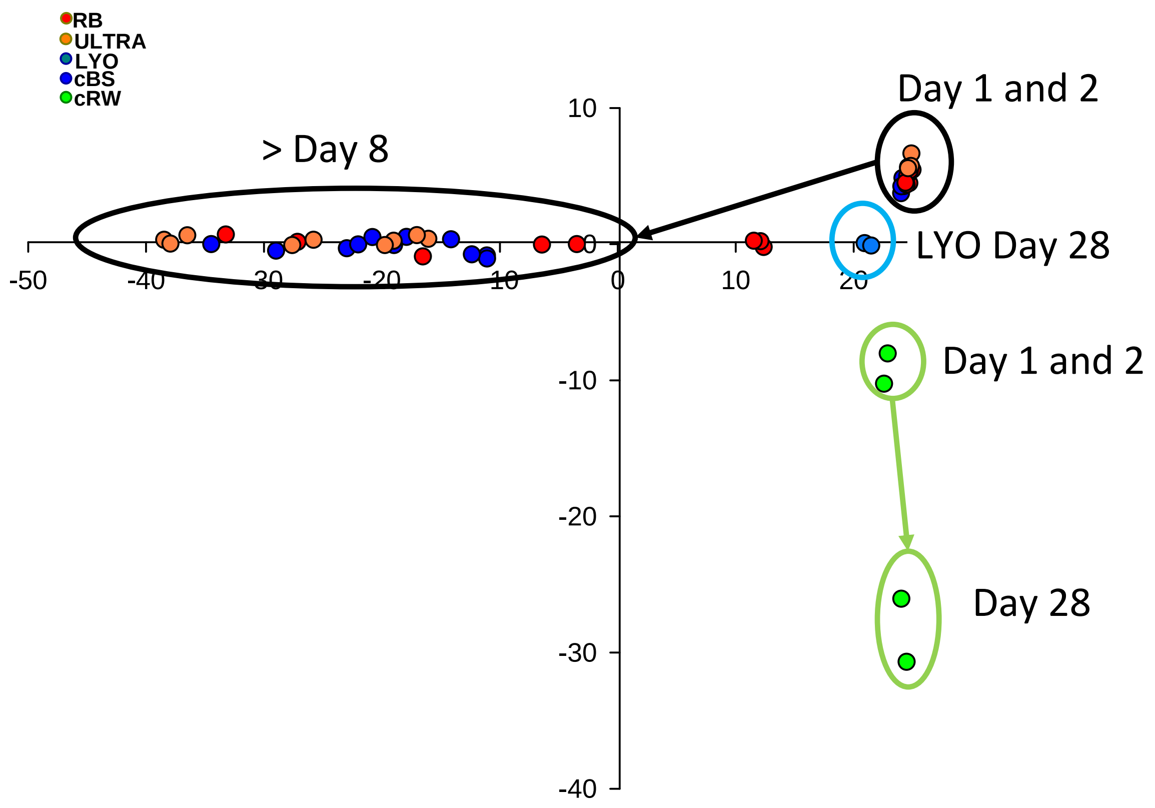

Figure S8. Principle component analysis (PCA) of changes in the operational taxonomic units (OTU) composition during the experiment. The PCA is basis for the OTU loadings in Figure S7. Eigenvalue PC1 = 80.6%; PC2 = 6.6 %; PC3 = 5.0. For abbreviation of the treatments see Fig 1.
