# Supplementary material for: Uncoupling of Bacterial and Terrigenous Dissolved Organic Matter Dynamics in Decomposition Experiments
Source: PLoS One. 2014 Apr 9;9(4):e93945. doi: 10.1371/journal.pone.0093945 (PMC3981725; doi:10.1371/journal.pone.0093945)
Supplement: Table S1 — Chemical composition of Artificial Sea Water. Solutions (A), (B) and (C) were separately prepared with ultrapure water. (PDF) [file pone.0093945.s009.pdf]

Table S1 Chemical composition of Artificial Sea Water (ASW). Solutions (A), (B) and (C) were separately prepared with ultrapure water.

A

Elements	(g L^{-1})
NaCl	19.45
$MgSO_4$	3.24

B

Elements	(g L^{-1})
$MgCl_2$ $6H_2O$	12.59
$CaCl_2$ $2H_2O$	2.38
KCl	0.55
$FeCl_3$ $6H_2O$	0.1099
KBr	0.08
$SrCl_2$	0.0572
H_3BO_3	0.022
NaF	0.0024

C

Elements	(g L^{-1})
Na_2HPO_4	0.008
NH_4NO_3	0.0016
