# Supplementary material for: Uncoupling of Bacterial and Terrigenous Dissolved Organic Matter Dynamics in Decomposition Experiments
Source: PLoS One. 2014 Apr 9;9(4):e93945. doi: 10.1371/journal.pone.0093945 (PMC3981725; doi:10.1371/journal.pone.0093945)
Supplement: Table S2 — Experimental set up and conditions of the mesocosms. Abbreviations for treatments as in Fig. 1, solutions A, B, C see Table S1. (PDF) [file pone.0093945.s010.pdf]

Table S2. Experimental set up and conditions of the mesocosms. Abbreviations for treatments as in Fig. 1, solutions A, B, C see Table S1.

	Volume (L)	(C) (µl)	(A) (ml)	(B) (ml)	Baltic Sea water (L)	Ultrapure water (L)	tDOM source	Salinity	pH
River water and Sea water (RB)	25	683	364	643	12	-	12 l River water	8.3±0.2	7.3
Sea water control (cBS)	25	-	-	-	25	-	-	8.1±0.1	8
Retentate and Sea water (ULTRA)	15	410	228	404	7.5	6.581	919 ml retentate	8.4±0.2	7.3
Lyophilisate and Sea water (LYO)	1.6	41	23	55	-	0.689	67 ml of lyphilisated DOM	-	-
River water control (cRW)	4	109	-	-	-	-	2 l filtered RW + 2 l unfiltered RW	0.1±0.01	7.9
