# Supplementary material for: Uncoupling of Bacterial and Terrigenous Dissolved Organic Matter Dynamics in Decomposition Experiments
Source: PLoS One. 2014 Apr 9;9(4):e93945. doi: 10.1371/journal.pone.0093945 (PMC3981725; doi:10.1371/journal.pone.0093945)
Supplement: Table S4 — Statistical analysis of the dissolved organic carbon (DOC) decomposition in the experiments. (A) DOC decomposition in the mesocosms (same abbreviations as in Fig. 1). (B) Based on the DOC reduction, no significant difference in a one-way ANOVA test can be determined. (PDF) [file pone.0093945.s012.pdf]

Table S4. Statistical analysis of the dissolved organic carbon DOC decomposition in the experiments. (A) DOC decomposition in the mesocosms (same abbreviations as in Fig. 1). (B) Based on the DOC reduction, no significant difference in a one-way ANOVA test can be determined.

A

	DOC (µM)		
	day 1	day 28	reduction
ULTRA 1	381	353	28
ULTRA 2	377	351	26
ULTRA 3	388	360	28
RB 1	299	268	32
RB 2	347	271	76
RB 3	315	269	47
cBS 1	342	309	33
cBS 2	317	307	10
cBS 3	342	309	33
cRW 1	403	370	33
cRW 2	399	nd	nd
cRW 3	401	370	31

B

	Sum of sqrs	df	Mean square	F	p(same)
Between groups:	1324.95	3	441.649	2.332	0.1606
Within groups:	1325.88	7	189.412		
Total:	2650.83	10			
