# Supplementary material for: Uncoupling of Bacterial and Terrigenous Dissolved Organic Matter Dynamics in Decomposition Experiments
Source: PLoS One. 2014 Apr 9;9(4):e93945. doi: 10.1371/journal.pone.0093945 (PMC3981725; doi:10.1371/journal.pone.0093945)
Supplement: Table S5 — Bacterial activity and bacterial growth efficiency. Bacterial activity estimated by [3H]-thymidine incorporation and oxygen consumption measured by a luminescence-based optical sensor (nd = not determined; same abbreviations as in Fig. 1). (PDF) [file pone.0093945.s013.pdf]

Table S5. Bacterial activity and bacterial growth efficiency. Bacterial activity estimated by [^{3}H]-thymidine incorporation and oxygen consumption measured by a luminescence-based optical sensor (nd= not determined; same abbreviations as in Fig. 1).

**1 day**	Thymidine incooperation (pmol $h^{-1}L^{-1}$)	Carbon-production (µg C $L^{-1}h^{-1}$)	Oxygen consumption (mg $L^{-1}d^{-1}$)	Bacterial carbon demand (µg C $L^{-1}h^{-1}$)	Bacterial growth efficiency
cBS1	114	4.3	0.05	6.09	0.70
cBS2	41	1.5	nd	nd	nd
cBS3	70	2.6	nd	nd	nd
RB1	38	1.4	0.01	nd	nd
RB2	23	0.9	0.08	3.79	0.23
RB3	66	2.5	nd	nd	nd
ULTRA1	39	1.5	0.32	13.19	0.11
ULTRA2	39	1.5	0.12	5.88	0.25
ULTRA3	34	1.3	nd	nd	nd
**6 days**					
cBS1	40	1.5	0.29	12.15	0.12
cBS2	32	1.2	0.11	5.23	0.23
cBS3	37	1.4	0.29	12.01	0.11
RB1	31	1.2	0.35	13.99	0.08
RB2	45	1.7	0.39	15.99	0.11
RB3	41	1.5	0.78	30.12	0.05
ULTRA1	28	1.1	0.18	7.67	0.14
ULTRA2	31	1.1	0.52	20.21	0.06
ULTRA3	23	0.9	0.43	16.62	0.05
**14 days**					
cBS1	10	0.4	1.47	54.27	0.01
cBS2	12	0.5	0.12	4.87	0.10
cBS3	11	0.4	nd	nd	nd
RB1	15	0.5	0.1	4.21	0.13
RB2	19	0.7	0.1	4.36	0.16
RB3	14	0.5	nd	nd	nd
ULTRA1	14	0.5	0.12	4.91	0.10
ULTRA2	18	0.7	0.23	9.12	0.08
ULTRA3	14	0.5			
**21 days**					
cBS1	19	0.7	1.31	48.74	0.01
cBS2	20	0.8	0.56	21.30	0.04
cBS3	22	0.8	0.57	21.73	0.04
RB1	27	1.0	0.2	8.35	0.12
RB2	27	1.0	0.1	4.67	0.21
RB3	23	0.9	nd	nd	nd
ULTRA1	31	1.2	0.26	10.70	0.11
ULTRA2	29	1.1	0.29	11.73	0.09
ULTRA3	26	1.0	0.16	6.86	0.14
**28 days**					
cBS1	nd	nd	nd	nd	nd
cBS2	40	1.5	0.1	5.16	0.29
cBS3	32	1.2	nd	nd	nd
RB1	27	1.0	0.44	17.16	0.06
RB2	22	0.8	0.38	14.77	0.06
RB3	28	1.0	0.29	11.67	0.09
ULTRA1	23	0.9	0.42	16.27	0.05
ULTRA2	15	0.6	0.25	9.73	0.06
ULTRA3	23	0.9	0.32	12.59	0.07
