# Supplementary material for: Uncoupling of Bacterial and Terrigenous Dissolved Organic Matter Dynamics in Decomposition Experiments
Source: PLoS One. 2014 Apr 9;9(4):e93945. doi: 10.1371/journal.pone.0093945 (PMC3981725; doi:10.1371/journal.pone.0093945)
Supplement: Table S6 — Nanoflagellate counts (ml−1) in the mesocosms. For abbreviations see Fig. 1. (PDF) [file pone.0093945.s014.pdf]

Table S6. Nanoflagellate counts (ml^{-1}) in the mesocosms. For abbreviations see Fig. 1.

	Day 1	Day 2	Day 8
cBS1	3043	2088	1909
cBS2	6205	2566	895
cBS3	5489	3162	1671
RB1	3938	835	1372
RB2	3162	1760	1611
RB3	3341	2715	1432
ULTRA1	2566	626	955
ULTRA2	2924	1820	2446
ULTRA3	2864	1551	2208
