# Supplementary material for: Uncoupling of Bacterial and Terrigenous Dissolved Organic Matter Dynamics in Decomposition Experiments
Source: PLoS One. 2014 Apr 9;9(4):e93945. doi: 10.1371/journal.pone.0093945 (PMC3981725; doi:10.1371/journal.pone.0093945)
Supplement: Table S7 — ANOSIM test of the dissolved organic matter composition. ANOSIM test of all day 0 and day 28 samples from the treatments based on Bray Curtis dissimilarity, calculated with PAST, (A) represent the R- values and (B) the uncorrected p-values. (PDF) [file pone.0093945.s015.pdf]

Table S7. ANOSIM test of the dissolved organic matter composition.
ANOSIM test of all day 0 and day 28 samples from the treatments based on Bray Curtis dissimilarity, calculated with PAST, (A) represent the R- values and (B) the uncorrected p-values.

A

	Day 0 LYO	Day 28 LYO	Day 0 cBS	Day 28 cBS	Day 0 cRW	Day 28 cRW	Day 0 RB	Day 28 RB	Day 0 Ultra
d28 LYO	0,3704								
d0 OST	1	1							
d28 OST	1	1	0,03704						
d0 cRW	0,7037	0,7778	1	1					
d28 cRW	1	1	1	1	-0,03704				
d0 RB	0,5556	0,7037	1	1	0,9259	1			
d28 RB	0,5833	1	1	1	0,8333	1	-0,3333		
d0 Ultra	1	1	1	1	0,5556	1	1	1	
d28 Ultra	1	1	1	1	0,5556	1	1	1	0,4444

B

	Day 28 LYO	Day 0 cBS	Day 28 cBS	Day 0 cRW	Day 28 cRW	Day 0 RB	Day 28 RB	Day 0 Ultra	Day 28 Ultra
d0 LYO	0,2003	0,0986	0,0988	0,0983	0,1031	0,208	0,2036	0,1019	0,0956
d28 LYO		0,098	0,1025	0,1006	0,1013	0,1	0,1036	0,1014	0,1
d0 OST			0,2968	0,1041	0,1004	0,1004	0,1033	0,098	0,0964
d28 OST				0,1036	0,1043	0,105	0,0991	0,0976	0,0986
d0 cRW					0,6024	0,0998	0,1011	0,1006	0,1027
d28 cRW						0,099	0,0952	0,1022	0,1036
d0 RB							1	0,1025	0,1037
d28 RB								0,0966	0,0992
d0 Ultra									0,0964
