# Supplementary material for: Uncoupling of Bacterial and Terrigenous Dissolved Organic Matter Dynamics in Decomposition Experiments
Source: PLoS One. 2014 Apr 9;9(4):e93945. doi: 10.1371/journal.pone.0093945 (PMC3981725; doi:10.1371/journal.pone.0093945)
Supplement: Table S8 — Loadings of a PCA analysis of the operational taxonomic units (OTUs) in the experiments. Loadings of a PCA analysis indicating OTUs contributing >0.05 or <0.05 to a principle component. (PDF) [file pone.0093945.s016.pdf]

Table S8. Loadings of a PCA analysis of the operational taxonomic units (OTUs) in the experiments. Loadings of a PCA analysis indicating OTUs contributing > 0.05 or < 0.05 to a principle component.

	Axis 1	Axis 2	Axis 3
(A) Perlucibaca OTU	0.9675	-0.02551	-0.1901
(B) Alteromonas OTU	0.1488	0.028	0.9284
12. Planctomyces OTU	0.02664	-0.009439	0.1461
11. LD29 OTU	0.002604	-0.05466	0.07214
17. Flavobacterium OTU	-0.008971	0.08051	-0.01981
5. Limnohabitans OTU	-0.009954	0.06035	-0.01147
1. GKS98 OTU	-0.0108	0.06063	-0.01045
19. HGC I OTU	-0.01088	0.07244	-0.009628
13. Variovorax OTU	-0.01174	0.07724	-0.01398
6. Aquabacterium OTU	-0.01221	0.1528	-0.02328
15. Gemmata OTU	-0.01297	0.0837	-0.002991
14. SAR11 OTU	-0.01847	-0.06628	-0.005947
2. Flectobacillus OTU	-0.01877	0.06214	-0.01332
7. Arcobacter OTU	-0.01951	-0.05798	-0.02835
22. SAR86 OTU	-0.02228	-0.06466	-0.01861
18. Flavobacterium OTU	-0.02372	-0.06623	-0.02811
24. Sediminibacterium OTU	-0.02389	0.1404	-0.007044
23. Polynucleobacter OTU	-0.02392	0.1839	-0.04068
25. SAR86 OTU	-0.02515	-0.1318	0.02489
10. Roseibacterium OTU	-0.0313	-0.08054	-0.02488
21. NS5 OTU	-0.03225	-0.1332	-0.0706
4. Roseobacter OTU	-0.03556	-0.09096	-0.0329
20. Fluviicola OTU	-0.04179	-0.1075	-0.02778
9. Synechococcus OTU	-0.04664	-0.1138	-0.02865
8. BAL58 OTU	-0.06792	-0.2094	-0.09361
16. Nautella OTU	-0.08009	-0.2034	-0.07552
3. Sufflavibacter OTU	-0.08117	-0.2528	-0.1186
